# A nanoengineered MSC therapeutic for synergistic therapy of lupus nephritis via cfDNA clearance and targeted immunomodulation

**DOI:** 10.7150/thno.136356

**Published:** 2026-09-03

**Authors:** Jiang Tian, Qinyao Xu, Zexin Wang, Zeyu Wang, Haofang Zhu, Lingyun Sun, Zhongwei Gu

**Affiliations:** 1 Department of Rheumatology and Immunology, Nanjing Drum Tower Hospital, Affiliated Hospital of Medical School, Nanjing University, Nanjing, China.; 2 Department of Rheumatology and Immunology, The First Affiliated Hospital of Anhui Medical University, Hefei, China.; 3 Department of Rheumatology and Immunology, The Second Affiliated Hospital of Anhui Medical University, Hefei, China.; 4 Research Institute for Biomaterials, Tech Institute for Advanced Materials, College of Materials Science and Engineering, Suqian Advanced Materials Industry Technology Innovation Center, Nanjing Tech University, Nanjing 211816, China.

**Keywords:** MSCs, peptide dendrimer, homing, cell-free DNA, lupus nephritis

## Abstract

**Rationale:**

Lupus nephritis (LN) remains a clinical challenge due to the dose-limiting toxicity of conventional immunosuppressants and the poor engraftment/efficacy of mesenchymal stromal cell (MSC) therapies.

**Methods:**

We report a novel nanoengineered MSC therapeutic for LN by engineering MSCs with a cell-free DNA (cfDNA)-targeting dendritic nanogel. To obtain the hybrid MSC-G3K@T system, we coupled tacrolimus-loaded, disulfide-crosslinked polylysine dendrimer nanogels (G3K@T) onto the MSC membrane. Our system exploits two synergistic targeting strategies: the innate homing capacity of MSCs, plus the specific affinity of the cationic G3K dendrimer for anionic cfDNA, which enriches in inflamed kidneys.

**Results:**

Our system acts through two coordinated actions: the nanogel responds to the oxidative renal microenvironment and releases tacrolimus in a spatiotemporally controlled manner, thereby suppressing T-cell activation. At the same time, it scavenges cfDNA, blocking TLR-mediated pro-inflammatory signaling. Working together with the inherent immunomodulatory functions of MSCs, this dual-action strategy yielded robust efficacy in MRL/lpr mice, including a significant reversal of T-cell dysregulation, restoration of glomerular integrity, and reduction of proteinuria and IgG deposition.

**Conclusions:**

We propose that MSC-G3K@T offers a promising route to augment cell-based therapies, creating a versatile platform that could be used to treat autoimmune and inflammatory diseases.

## Introduction

In systemic lupus erythematosus (SLE), lupus nephritis (LN) occurs in 40-60% of patients, representing the most serious organ manifestation and a leading contributor to end-stage renal disease 1-7. Although tacrolimus (FK506) suppresses T-cell activity and partly improves kidney function, its poor tissue targeting and fast elimination force the use of high systemic doses, which frequently cause serious toxicities like nephrotoxicity and infection 8-12. Mesenchymal stromal cell (MSC) treatment is an attractive option because it naturally regulates immune function and promotes tissue repair 13-19. Yet, translating MSC therapy to the clinic remains challenging due to poor engraftment, immune rejection by the host, and inefficient homing to target organs 20-24. Therefore, a multifunctional strategy is urgently needed to boost MSC survival, broaden their therapeutic actions, and achieve site-specific drug release.

To tackle these problems, we engineered a dendritic polylysine nanogel platform that turns MSCs into dual-function therapeutics for LN. Previous strategies like genetic modification or hydrogel encapsulation improved cell retention, but often compromised their innate migration ability or failed to provide on-demand drug release 25-30. In contrast, peptide dendrimers enable controllable drug loading, possess multivalent surface groups, and react to ROS. Most importantly, they bind cell-free DNA (cfDNA) tightly, as cfDNA accumulates in inflamed kidneys 31-36. The G3K dendrimer binds anionic cfDNA and homes to the kidneys, while also blocking cfDNA-triggered TLR signaling to provide intrinsic anti-inflammatory activity 37-40. While dendritic polymers are widely used as nanocarriers, combining them with living MSCs to create a single, cooperative therapeutic platform remains rare, particularly for LN.

In this work, we created a nanogel-engineered MSC, named MSC-G3K@T, by attaching tacrolimus-loaded, disulfide-crosslinked G3K nanogels to the cell surface through DSPE anchors (**Figure 1**). Our platform exploits oxidative conditions in LN kidneys to trigger drug release via ROS. Meanwhile, it homes to the kidneys more efficiently by combining two targeting routes: the natural migration of MSCs toward inflamed tissue and the cfDNA-binding ability of the conjugated G3K nanogel. In addition to site-specific delivery, the G3K nanogel removes cfDNA from renal tissue, thereby blocking TLR signaling and the ensuing inflammatory cascade. Together with tacrolimus-mediated T-cell suppression, the intrinsic immunomodulatory functions of MSCs reversed T-cell dysregulation, preserved glomerular structure, and reduced proteinuria and IgG deposition in MRL/lpr mice. Unlike earlier methods that simply encapsulate MSCs or electrostatically adsorb nanogels, our strategy features two main innovations: (i) DSPE-PEG lipid insertion yields a more uniform, durable nanogel coating, preserves membrane integrity, and avoids early internalization; (ii) ROS-triggered drug release does not impair MSC viability or their immunomodulatory functions, representing a clear improvement over passive encapsulation. Beyond LN treatment, this study establishes a versatile nanoengineered MSC therapeutic that merges synthetic nanocarriers with cellular therapies, opening avenues for treating a spectrum of autoimmune and inflammatory diseases.

## Materials and Methods

### Materials

The following reagents were sourced from Aladdin Co., Ltd.: (3-aminopropyl)-triethoxysilane, Boc-Lys(Boc)-OH, HBTU, HOBt, and DIPEA. MedChemExpress provided DSP crosslinker, Tacrolimus (TAC), and CXCL12. Ponsure Biotechnology Co., Ltd. supplied the DSPE-PEG-NHS conjugate. Gibco delivered DMEM/F12, RPMI 1640, IMDM, DMEM, and FBS. Both forms of CpG 2006 (unconjugated and Cy5.5-conjugated) arrived from Genscript. Yeasen Biotechnology Co., Ltd. provided calf thymus DNA solution. Ruixi Biotechnology Co., Ltd. handled the custom fluorescent labeling of tacrolimus with Cy5. Cell proliferation dye eFluor 670 was ordered from eBioscience. Flow cytometry was performed with the following antibodies, all from BD Biosciences: Fixable Viability Stain 780, CD4, CD25, FOXP3, PD-1, CXCR5, IL-17A, CD11B, F4/80, CD86, CD206, and LY-6G. C3, IgG, and Goat anti-rabbit IgG (Alexa Fluor 750) were obtained from Abcam. Invitrogen supplied CellMask orange plasma membrane stain, goat anti-mouse 568 and goat anti-rabbit 488. Thermo Scientific provided NHS-Fluorescein.

### Synthesis and functionalization of G3KDSP

To synthesize G3KDSP nanogels, G3K with a polyhedral oligomeric silsesquioxane (POSS) core was prepared as described previously. G3KDSP nanogels were prepared via crosslinking between G3-Lys and DSP crosslinker in DMF solvent. Three G3-Lys/DSP molar ratios (1:2, 1:4, and 1:8) were tested to optimize the synthetic conditions. Briefly, 10 mL of DMF solutions containing graded doses of DSP crosslinker were slowly introduced into 50 mL DMF solution containing 500 mg of G3-Lys. Stirring was maintained at 1500 rpm under ambient conditions for 24 hours. The solution was fully dialyzed against DMF and deionized water using 1000 Da MWCO dialysis membranes (Spectrum). After lyophilization, the harvested white flocculent nanogels were preserved under vacuum. Confirmation of successful G3KDSP nanogel synthesis was achieved through ^1^H NMR spectroscopy.

### Synthesis and functionalization of G3K@T-DSPE

A 10 mL DMF solution of 100 mg DSP was slowly dropped into 50 mL DMF solution containing 500 mg G3K and 50 mg TAC. Stirring was maintained at 1500 rpm for 24 hours. Subsequently, 100 mg DSPE-PEG-NHS was then introduced, and the reaction proceeded for another 24 hours. The obtained solution was fully dialyzed with 1000 Da MWCO dialysis membranes (Spectrum) using DMF and deionized water. The TAC loading capacity of G3K@T-DSPE was quantified at 220 nm in PBS via a TECAN Spark microplate reader. We quantified drug loading content and efficiency using the equations below: DLC (%) = (mass of TAC encapsulated / total mass of G3K@T-DSPE) × 100%; DLE (%) = (mass of TAC encapsulated / initial mass of TAC fed) × 100%.

### *In vitro* TAC release of G3K@T

To conduct the *in vitro* TAC release assay, G3K@T was prepared using the method described above. 5 mg of G3K@T was dispersed in 1 mL PBS (pH 7.4) and transferred into a dialysis bag with a 1000 Da MWCO (Spectrum). We placed the dialysis bag into 20 mL of PBS or 1 μM H₂O₂ as the release medium and shook gently at 70 rpm under 37 °C. At scheduled time points, 200 µL release medium was withdrawn for detection, with the same volume of fresh buffer replenished each time. The TAC concentration in collected liquid was tested at 220 nm via a TECAN Spark microplate reader with standard curve calibration.

### Cell culture

Mesenchymal stromal cells (MSCs) were isolated from fresh human adipose tissue and expanded in DMEM/F12. RAW 264.7 macrophages were maintained in DMEM medium. IMDM was used to culture Ramos Blue™ reporter cells (Invitrogen). CD4⁺ T cells were cultivated in RPMI-1640 under the same culture environment. Murine CD4⁺ T lymphocytes were separated from the spleens of BALB/c mice.

### Fabrication and characterization of MSC-G3K@T

MSC-G3K@T was generated by co-incubating MSCs with G3K@T-DSPE. In brief, MSCs were plated in 10 cm dishes at 5 × 10⁶ cells per dish. G3K@T-DSPE was introduced into the medium to reach a final tacrolimus concentration of 10 μM. The mixture was then incubated at 37 °C for 1-8 hours. Cells were harvested (350 g, 5 min) to remove unbound material. Cytotoxicity was evaluated with a CCK-8 assay: MSCs (2 × 10⁴ /well, 96-well plate) were incubated for 24 hours, then exposed to G3K@T-DSPE at different tacrolimus levels for an additional 24 hours. Viability was determined from A450. Loading capacity was optimized by quantifying tacrolimus via high-performance liquid chromatography (HPLC) after varying incubation conditions.

Physical properties including size, surface charge, and morphology were characterized with a NS-90Z Plus nanoparticle analyzer and transmission electron microscopy (HT7800, Hitachi). The surface distribution of nanogels on MSCs was visualized by scanning electron microscopy (SU8100, Hitachi). Ultraviolet–visible absorption spectra were acquired with a microplate reader (Spark, TECAN). Conjugation of MSCs and G3K@T-DSPE was further validated by CLSM (STELLARIS STED, Leica) and FCM (DxP Athena, Cytek). In brief, MSCs were grown on confocal dishes (1 × 10⁵ cells) to approximately 90% confluence, labeled with CellMask orange, and then incubated with G3K@T-DSPE (FITC-G3K, Cy5-TAC) for 2 hours. In addition, MSCs were incubated with G3K@T for 1, 2, and 4 hours with or without DSPE-PEG-NHS modification prior to imaging.

### Agarose gel electrophoresis

CpG 2006 solutions were mixed with G3KDSP, MSCs, or MSC-G3K@T to obtain a final CpG concentration of 25 μg/mL in each complex. Samples were kept at 4 °C for 4 hours and were loaded onto 1% agarose gels pre-stained with Gel-Red (Beyotime) and run at 90 V for 25 min. Uncomplexed CpG 2006 served as a control. Gels were imaged with a Gelview 5000 pro II system (Biolight Biotechnology).

### Binding efficiency of MSC-G3K@T and cfDNA

5 μL of calf thymus DNA (1 mg/mL in PBS) was premixed with 5 μL EtBr solution (0.5 mg/mL in PBS). The test substance (equivalent to 4 μL G3KDSP at 1 mg/mL) was introduced and total volume was adjusted to 200 μL with FBS or PBS. Incubation (24 hours) was followed by transferring 100 μL supernatant to a 96-well plate and recording fluorescence (ex 485 nm / em 590 nm). Binding efficiency = (1 - (A - A₀) / (A₁ - A₀)) × 100%, with A = EtBr/DNA fluorescence after treatment, A₀ = EtBr only, A₁ = EtBr/DNA without treatment.

### Inhibition of DNA uptake by cells

Following a 1-h incubation of MSCs (4.5×10^6^ /well) with FITC-labeled G3K-DSPE@T nanogels, unattached nanogels were removed by centrifugation, and then cells were exposed to Cy5-CpG for 1–4 hours. After CellMask Orange staining (10 min), images were obtained by CLSM. For competition assays, RAW264.7 macrophages (5×10^4^ cells) on confocal dishes were treated with 1 μM Cy5-CpG 2006 and 1 μM MSC-G3K@T (FITC-G3K, CellMask orange-labeled membrane) for 1, 2, and 4 hours. Hoechst (10 min) was applied before CLSM.

### Inhibition of TLR signaling pathway

Ramos Blue^TM^ reporter cells (5×10^4^) were stimulated with 1 μM CpG 2006 together with various concentrations of G3KDSP. Supernatants were harvested 24 hours later, and SEAP activity was assessed using QUANTI-Blue (InvivoGen) following the supplied protocol, with detection at 650 nm. TLR activation (%) was calculated using the formula (X - X₀)/(X₁ - X₀) × 100%, where X is the sample OD, X₀ is the untreated control, and X₁ is the agonist-only group.

To compare materials, Pam3CSK4 (1 μM) or CpG 2006 (1 μM) was incubated with each preparation (equivalent to 25 μg/mL G3KDSP) and then added to Ramos Blue™ cells. After 24 hours, SEAP activity was quantified as above. In RAW264.7 macrophages, 1 μM CpG 2006 was applied together with 1 μM of each material. TNF-α and IL-6 levels in the 48-hour supernatants were measured by ELISA (FineTest).

### Western blotting

Macrophages were lysed and total protein was quantified with a BCA kit (Beyotime). Protein samples were denatured for 15 min at 95 °C in loading buffer, separated on 10% SDS-PAGE gels (120 V, 90 minutes), and transferred to PVDF membranes (220 mA, 80 min). Blocking was carried out with 5% skimmed milk for 90 minutes, followed by overnight probing at 4 °C with the following primary antibodies (each 1:5000): anti-p65, anti-p-p65, anti-MyD88, and anti-β-actin. Membranes were washed three times with TBST and then incubated with goat anti-rabbit IgG (1:2000) for 2 hours. Signals were developed with ECL reagent (Servicebio) and captured using a Tanon chemiluminescence imager.

### *In vitro* migration of MSC-G3K@T

Transwell chambers (8.0 μm pores) were used to assess migration. MSCs or MSC-G3K@T were placed in the upper compartment and incubated for 24 hours, after which CXCL12 (100 ng/mL) was supplied to the lower well. Following 24 h of further migration, the upper-chamber cells were dyed with 0.1% crystal violet, destained using 33% acetic acid, and quantified by absorbance at 590 nm.

### qPCR

We isolated total RNA from both MSC-G3K@T and naïve MSCs with an RNA isolation kit (FastPure V2, Vazyme). Subsequent cDNA synthesis used a reverse transcription kit (HiScript III, Vazyme), and qPCR was carried out with Master Mix (ChamQ, Vazyme) to measure *CXCR4*, *IL-10*, *TGF-β*, *PTGES*, and *GAPDH*. For T-cell experiments, CD4⁺ cells from different treatment groups were analyzed for *RORγt* and *FOXP3* expression using the same protocol.

### *In vitro* T cell culture

For T-cell activation, 24-well plates were first coated with anti-CD3 (2 μg/mL, Elabscience). Mouse CD4⁺ T cells were then added at 5 × 10⁵ cells/well and maintained in RPMI-1640 containing anti-CD28 (2 μg/mL, Elabscience). For T helper 17 (T_H_17) cells polarization, cultures were supplemented with IL-6 (20 ng/mL, Novoprotein) and TGF-β1 (2 ng/mL, Novoprotein). For regulatory T (Treg) cells differentiation, TGF-β1 (5 ng/mL, Novoprotein) and IL-2 (100 U/mL, Novoprotein) were added.

### Cell proliferation evaluation

Mouse CD4⁺ T lymphocytes were incubated with the eFluor 670 proliferation dye (2 μM, eBioscience) for 20 min and washed. Labeled cells were co-cultured with the indicated treatments (PBS, G3KDSP, MSCs, tacrolimus, MSC-G3K, or MSC-G3K@T) at a 10:1 T-cell-to-MSC ratio under anti-CD28/anti-CD3 (2 μg/mL). After 5 days, proliferation was assessed by flow cytometry.

### Flow cytometry

Frequencies of T_H_17 and Treg were measured with flow cytometry (Aurora, Cytek). For the co-culture setup, CD4⁺ T cells (2 × 10⁵ cells/well) were plated in the lower chamber and co-cultured for 3 days with the following additions to the upper chamber: G3KDSP (0.6 μM), MSCs (2 × 10^4^ cells), TAC (0.5 μM), MSC-G3K (2 × 10^4^ cells) and MSC-G3K@T (2 × 10^4^ cells). For T_H_17 staining, cells were restimulated with Leukocyte Activation Cocktail (PMA/Ionomycin/BFA) for 4 hours, blocked with Mouse Fc Block, and stained with Fixable Viability Stain 780 and APC anti-CD4. After fixation/permeabilization, cells were labeled with Brilliant Violet 421 anti-IL-17A. Tregs were identified by surface staining with APC anti-CD4 and BB515 anti-CD25, followed by fixation/permeabilization and intracellular staining with PE anti-FOXP3. All reagents were obtained from BD Biosciences. The detailed gating strategies are shown in **Figure S30**.

### ELISA

We measured IL-10 and IL-17A in CD4⁺ T-cell supernatants using mouse ELISA kits (FineTest) according to the supplier’s protocol. Samples were assayed in quintuplicate alongside kit standards.

### Biodistribution of materials

Cy5-labeled TAC, DiD-labeled MSCs, and Cy5/DiD-labeled MSC-G3K@T were injected intravenously into MRL/lpr mice. For DiD labeling, MSC-G3K@T (100 µg in 1 mL) was incubated with 1 µL of 1 mM DiD (Vybrant™) at 37 °C for 30 minutes, and then ultracentrifuged (120,000 × g, 70 minutes) to remove the unbound dye. Fluorescence distribution was monitored with an AniView Pro *in vivo* imaging system (Biolight Biotechnology) at 2, 4, 24, 48 and 72 hours. Three animals per time point were euthanized. *Ex vivo* imaging and ROI quantification (AniView) were carried out on the excised lymph nodes and major organs. Organs were also cryosectioned (HM525 NX, Epredia), stained with DAPI, and examined by CLSM. Renal infiltration of CD4⁺ T cells and macrophages was evaluated by immunofluorescence. Drug release in the kidney was visualized using triple-labeled MSC-G3K@T (CellMask Orange-MSC, FITC-G3K, Cy5-TAC).

### *In vivo* assessment of SLE therapy

All procedures were approved by the Drum Tower Hospital Animal Welfare Ethics Committee (No. 2021AE01008). We utilized 8-10-week-old male MRL/lpr mice (SPF, Cavens) that spontaneously develop lupus. Forty-eight mice were allocated to six groups (n = 8). From week 19 to 22, mice received weekly injections via tail vein with 100 μL of 0.9% NaCl, G3KDSP, MSCs, TAC, MSC-G3K, or MSC-G3K@T. Body weight and proteinuria were recorded weekly. Serum creatinine and BUN were measured with commercial kits (Jiancheng Bioengineering). Serum cfDNA was also determined. Anti-dsDNA antibodies were quantified by ELISA (ZCIBIO Technology).

### Immunohistochemistry

Kidney specimens were formalin-fixed, paraffin-embedded, sectioned, and subsequently stained with HE, Masson’s trichrome, and PAS. Histopathological lesions were scored by two independent renal pathologists on a 0–4 scale (1: 1–25%, 2: 26–50%, 3: 51–75%, 4: > 75% injury). For immunofluorescence, cryosections (10 μm) of OCT-embedded kidneys were subjected to fixation, Triton X-100 permeabilization, and 10% FBS blocking. Immunostaining was performed using anti-mouse IgG or anti-mouse C3 antibodies (Abcam), counterstained with DAPI, and imaged by CLSM (STELLARIS STED, Leica). Fluorescence intensities were quantified using ImageJ.

### Safety evaluation

Six groups of healthy mice (n = 5 per group) were given a once-weekly intravenous injection of 0.9% NaCl, G3KDSP, MSCs, TAC, MSC-G3K, or MSC-G3K@T (all at 1 mg/kg equivalent dose). At week 4, serum and major organs were collected. Liver and kidney function parameters were measured. Tissues were fixed, processed for H&E staining, and scanned with a KF-PRO-020 digital slide scanner (KFBIO) for histological assessment.

### Immune profile analysis in MRL/lpr mouse spleens

After the final treatment, spleens and kidneys were harvested and processed into single-cell suspensions. T_H_17 (CD4⁺IL-17A⁺), Treg (CD4⁺CD25⁺FOXP3⁺) and Tfh (CD4⁺PD-1⁺CXCR5⁺) populations were analyzed by intracellular and nuclear staining. Neutrophils (CD11b⁺Ly-6G⁺) and macrophage subsets were examined by surface staining and flow cytometry. M2 was defined as F4/80⁺CD11b⁺CD206⁺, and M1 as F4/80⁺CD11b⁺CD86⁺.

### Statistics and reproducibility

At least three biological replicates were used for all quantitative experiments. Group differences were evaluated using one-way analysis of variance (ANOVA) followed by Tukey’s post-hoc test. Significance was accepted at p < 0.05.

## Results and Discussion

### Design, fabrication and characterization of MSC-G3K@T

We engineered a cationic nanogel-based cfDNA scavenger using generation 3 poly(L-lysine) dendrimers built around a polyhedral oligomeric silsesquioxane (POSS) core (denoted G3K), synthesized as previously reported 41. To confer redox-responsiveness, the hydrophilic G3K dendrimers were crosslinked via disulfide bonds using DSP crosslinker, yielding G3KDSP nanogels. This strategic design ensures that the nanogels remain stable during systemic circulation but undergo rapid disintegration upon encountering the heightened oxidative stress within inflamed renal tissues, facilitating on-demand payload release. The synthesis and structural confirmation of G3K and G3KDSP nanogels are detailed in **Figures S1 and S2**. By optimizing the molar ratio of G3K to DSP crosslinker, we determined that a 1:4 ratio produced G3KDSP nanogels with an ideal balance of yield, hydrodynamic size, and zeta potential (**Figure S3**).

Subsequently, the immunosuppressant tacrolimus was efficiently encapsulated during the crosslinking process to form drug-loaded G3K@T nanogels. The resulting G3K@T nanogels exhibited a uniform particle size of 201.54 ± 65.26 nm, slightly larger than the G3KDSP nanogels (173.09 ± 50.84 nm). The measured drug loading content (DLC) reached 7.46%, alongside a loading efficiency (DLE) of 93.56% (**Figure S4**). In addition, the nanogel formulations showed favorable colloidal stability within pH 7.4 PBS (**Figure S5**). To enable stable anchorage to cell membranes, we reacted DSPE-PEG-NHS with a fraction of surface amino moieties of G3K dendrimers, generating G3K-DSPE@T nanogel products. ¹H nuclear magnetic resonance (¹H NMR) characterization verified successful DSPE-PEG grafting, with a grafting ratio of 6.88% (**Figure S6**). For clarity and simplicity in subsequent discussions, the DSPE-PEG-modified, drug-loaded nanogel (G3K-DSPE@T) is hereafter referred to as G3K@T nanogel.

The DSPE-PEG-modified G3K@T nanogels were then efficiently conjugated onto MSC membranes via physical insertion, forming our core therapeutic platform: MSC-G3K@T. This engineering process maintained high cell viability (>80%) across various nanogel concentrations over 24 hours (**Figure S7A**). To further verify that the cationic nanogel conjugation does not disrupt membrane integrity, we performed a lactate dehydrogenase (LDH) release assay. MSCs incubated with G3K@T nanogels at various concentrations for 24 hours showed no obvious elevation of LDH leakage relative to untreated groups (**Figure S7B**), confirming that the DSPE-mediated membrane anchoring strategy preserves MSC membrane integrity. Additionally, the surface marker profile characteristic of MSC was assessed by flow cytometry. MSC-G3K@T maintained high expression of CD105 and CD90 (> 99%), with negligible expression of CD34 and CD45 (< 1%) (**Figure S8**). This result indicates that the DSPE-mediated membrane anchoring strategy preserves the canonical MSC immunophenotype, consistent with the observed retention of multilineage differentiation capacity.

After 2-hour incubation with G3K@T nanogels at a tacrolimus concentration of 10 μM, the final DLC of single MSC-G3K@T cells was measured to be 34.45 ± 0.55 pg (**Figure S9**). Effective cell surface modification was fully verified by a distinct zeta potential reversal. The potential shifted from -17.83 mV for native MSCs to over +17.32 mV for MSC-G3K@T, indicating the formation of a dense, cationic nanogel corona (**Figure 2A-B**). Scanning electron microscopy (SEM) further confirmed the robust attachment of numerous spherical nanogels onto the MSC membrane (**Figure 2C**). The co-localization of G3K, tacrolimus, and MSCs was corroborated by confocal laser scanning microscopy (CLSM) and UV absorbance spectroscopy (**Figure 2D-E**). Moreover, the DSPE-PEG coating allowed the nanogels to remain on the cell membrane with minimal internalization for at least 4 hours, while non-PEGylated G3K@T was rapidly taken up (**Figure S10**). Flow cytometry further verified the successful conjugation: DiO-labeled MSCs and FITC-labeled G3K@T appeared mostly in the quadrant gated for dual positivity (**Figure 2F**). Collectively, these data indicate effective surface modification of MSCs.

Our system is engineered to respond to the oxidative environment of diseased LN kidneys. In inflamed kidneys, high ROS levels break the disulfide crosslinks of G3K@T nanogels, causing them to disassemble and release tacrolimus in a controlled manner (**Figure 2G**). We incubated the nanogels with 1 mM H₂O₂ for 48 hours and found this markedly destabilized their architecture. The nanogels then disassembled into dispersed G3K dendrimer units, and the hydrodynamic diameter rose to around 450 nm (**Figure 2H**). The release data confirmed this stimuli-responsive behavior: under physiological conditions (PBS, pH 7.4), tacrolimus was released slowly and steadily, reaching only about 50% cumulative release after 120 hours. Drug release reached about 90% upon exposure to 1 mM H₂O₂, a stimulus that mimics disease-related oxidative stress, nearly double the level in the blank group over the same incubation period (**Figure 2I**). This difference in release kinetics allows the drug to act mainly at the disease site, with minimal off-target effects.

### cfDNA scavenging and TLR signaling inhibition by MSC-G3K@T

Beyond its drug delivery function, the G3K component was validated as a potent scavenger for cfDNA, a key damage-associated molecular pattern in LN. A gel retardation assay confirmed that G3KDSP nanogels and MSC-G3K@T completely bound CpG-ODN2006 (a cfDNA mimic), leaving no free DNA detectable (**Figure S11**). This binding affinity was quantitatively assessed through ethidium bromide (EtBr)-based competitive binding analysis. G3KDSP nanogels, MSC-G3K (without tacrolimus encapsulation), and MSC-G3K@T all exhibited high DNA binding efficiency in pH 7.4 PBS as well as serum-supplied media. Notably, this scavenging capability persisted even after the ROS-triggered disintegration of the nanogels, as demonstrated by MSC-G3K@T pre-incubated with H₂O₂. In contrast, native MSCs showed negligible DNA binding due to their anionic membrane (**Figure 2J**). These results confirm that the cationic nanogels’ cfDNA-neutralizing function is robust and persists in biologically relevant media, unimpeded by potential protein corona formation, highlighting its therapeutic potential for mitigating cfDNA-driven inflammation *in vivo*.

Confocal microscopy was used to track the interaction between cfDNA and MSC-G3K@T. The construct efficiently captured Quasar 670-labeled CpG-ODN2006, and membrane-associated cfDNA increased markedly over time. By 4 hours, pronounced accumulation was evident (**Figure 3A**). These images suggest that the cationic nanogel corona on the MSC surface functions like a “molecular sponge” for extracellular cfDNA, capturing it with high affinity in a time-dependent manner. This time-dependent accumulation further implies a stable scavenging function capable of supporting sustained therapy *in vivo*.

Further studies were conducted to explore whether MSC-G3K@T can suppress TLR activation via competitive neutralization of cfDNA by cationic G3KDSP nanogels. In Ramos Blue™ reporter cell assay, G3KDSP nanogels, MSC-G3K, and MSC-G3K@T significantly suppressed TLR9 activation induced by CpG-ODN2006, whereas native MSCs did not (**Figure 3B**). This result confirms that the TLR9 inhibition is specifically mediated by the cationic G3K component, not by the MSCs themselves. Specificity was confirmed, as none of the treatments affected TLR2 activation by Pam3CSK4, ruling out non-specific cytotoxic or broad immunosuppressive effects and highlighting the precision of this nucleic acid-targeting mechanism.

To further establish the tunability and therapeutic relevance of this effect, we evaluated TLR9 inhibition using MSC-G3K@T engineered with varying densities of surface-conjugated G3K@T nanogels. The inhibition exhibited a clear dose-dependency: MSC-G3K@T fabricated with a high conjugation dose (50 µg/mL nanogel protein equivalent) nearly completely abrogated TLR9 signaling, whereas a lower dose (5 µg/mL) reduced signaling to approximately 70% of the control level (**Figure S12**). This dose-response relationship not only validates the specificity of the effect but also demonstrates that the functional output of the engineered platform can be precisely modulated by the density of the nanogel corona.

To elucidate the downstream anti-inflammatory mechanism, we investigated whether the extracellular cfDNA sequestration by MSC-G3K@T could competitively inhibit its uptake by innate immune cells. Upon 4 hours co-culture of RAW264.7 macrophages and Quasar 670-conjugated CpG without additional treatments, confocal microscopy revealed robust intracellular accumulation of the ligand within endolysosomes, indicating efficient cellular internalization and TLR9 engagement (**Figure 3C**, top row). In stark contrast, when the same amount of CpG was co-incubated with both MSC-G3K@T and RAW264.7 cells, a markedly different distribution was observed over time. The fluorescent CpG was progressively and efficiently captured by the cationic nanogel corona on the MSC membrane, with the binding amount increasing from 1 to 4 hours. Consequently, only a minimal signal was detected inside the RAW264.7 cells at all time points (**Figure 3C**, bottom row). This visual evidence directly demonstrates that MSC-G3K@T acts as a high-affinity extracellular “sink”, outcompeting macrophages for cfDNA binding and thereby preventing its internalization and subsequent TLR recognition.

To directly link cfDNA scavenging to suppression of the TLR9 signaling cascade, we performed Western blot detection of p-p65, p65, and MyD88 in CpG-stimulated RAW264.7 cells. MSC-G3K@T, MSC-G3K, and free G3KDSP nanogels all markedly reduced p-p65 levels compared to the CpG-only group (**Figure 3D-E**), providing direct molecular evidence that nanogel-mediated cfDNA sequestration blunts the TLR9/MyD88/NF-κB axis. Consistent with this, the overexpression of proinflammatory IL-6 and TNF-α observed in CpG-only group was markedly inhibited after MSC-G3K@T treatment (**Figure 3F-G**). Native MSCs, lacking the cfDNA-scavenging capability, showed minimal inhibitory effects.

### Nano-engineering enhances the immunomodulatory phenotype of MSCs

To elucidate the global impact of nano-engineering on MSC functionality, we first performed transcriptome sequencing (RNA-seq) on MSC-G3K@T versus unconjugated naïve MSCs. Differential expression analysis revealed profound transcriptional reprogramming, with 994 genes upregulated and 1272 downregulated in the engineered cells (**Figure 4A**). GO enrichment analysis of the differentially expressed genes (DEGs) reflected this functional shift (**Figure 4B**). Within biological processes (BP), the DEGs pointed to immune regulatory activities, covering “T cell proliferation”, “production of molecular mediator of immune response”, “canonical NF-κB signal transduction”, and “activation of innate immune response”. This profile aligns with an immune-focused transcriptional shift in MSC-G3K@T. Cellular component (CC) analysis revealed shifts in “lysosomal lumen”, “autophagosome”, and “nuclear speck”, hinting at an adaptive reorganization of intracellular compartments that may aid survival under inflammatory conditions. In the molecular function (MF) category, enriched terms included “chromatin DNA binding”, “cytokine activity”, “cytokine receptor binding”, and “nuclease activity”. These enrichments suggest an enhanced ability to handle both immunomodulation and nucleic acid interactions. The latter function, in particular, aligns with the cfDNA-scavenging role of the conjugated nanogel.

To uncover the signaling pathways underlying this shift, we next employed Gene Set Enrichment Analysis (GSEA). MSC-G3K@T showed significant enrichment in four pivotal pathways. The robust activation of the TGF-β signaling pathway—central to inducing immune tolerance and tissue repair—and the JAK-STAT signaling pathway—critical for amplifying anti-inflammatory cytokine signaling—collectively underscore enhanced immunosuppressive capacity (**Figure 4C**). Concurrently, the marked upregulation of the chemokine signaling pathway provides a direct molecular basis for improved chemotaxis and homing capacity to sites of injury (**Figure S13A**). Equally significant was the enrichment of arachidonic acid metabolism, which indicates an enhanced biosynthetic potential for lipid mediators such as prostaglandin E2 (PGE2), key paracrine factors that mediate the resolution of inflammation and immunoregulation (**Figure S13B**).

The functional alterations described above received further support from Kyoto Encyclopedia of Genes and Genomes (KEGG) analysis. DEGs exhibited significant enrichment in multiple signaling pathways that maintain immune homeostasis and drive the polarization of immune cells toward regulatory phenotypes, including the Toll-like receptor (TLR), TGF-β, NF-κB, and JAK-STAT pathways (**Figure 4D**). Enrichment for “Chemokine signaling pathway” and “Cytokine-cytokine receptor interaction” adds further support for an immunomodulatory shift in the nanoengineered MSCs.

A heatmap of key immunomodulatory genes revealed distinct expression patterns between groups (**Figure 4E**). MSC-G3K@T showed clear upregulation of several immunomodulatory genes. These included *CXCR4*, which is linked to improved homing, as well as *IL-10*, *TGF-β*, and *PTGES*, all associated with anti-inflammatory responses. The NF-κB pathway inhibitor* NFKBIA* was also upregulated. Pro-inflammatory mediators, including *CXCL1* and *CSF3*, were downregulated in parallel. This reciprocal expression profile indicates a shift toward an anti-inflammatory, homing-competent phenotype, as confirmed by subsequent functional assays.

qPCR confirmed the upregulation of several key genes identified in the transcriptomic analysis. These included the homing receptor *CXCR4*, the anti-inflammatory mediators *TGF-β1* and* IL-10*, and *PTGES*, an enzyme responsible for *PGE2* synthesis (**Figure 4F**). To test whether excess cfDNA would compromise the immunomodulatory phenotype, we exposed MSC-G3K@T to CpG, a surrogate for pathological cfDNA. The expression of key immunomodulatory genes remained high and did not differ significantly from the unexposed MSC-G3K@T group. This control experiment shows that scavenging cfDNA does not impair the engineered MSCs’ own immunomodulatory capacity. Instead, it confirms that the cell’s therapeutic “voice” (its anti-inflammatory gene expression) remains potent even while it actively “silences” a pathogenic signal (cfDNA). More importantly, the molecular reprogramming translated directly into enhanced cellular function. In a Transwell assay, MSC-G3K@T demonstrated significantly increased migration toward the chemokine CXCL12 compared to naïve MSCs (**Figure 4G and Figure S14**), confirming the functional upregulation of the *CXCR4* signaling pathway observed in the GSEA.

Collectively, these results demonstrate that our nano-engineering strategy not only decorates the MSC surface but also induces transcriptomic changes that enhance homing capability (via *CXCR4*), anti-inflammatory cytokine release (via *IL-10*/*TGF-β*), and pro-resolving mediator synthesis (via *PTGES*/*PGE2*). The enhanced anti-inflammatory phenotype may arise from the cationic polylysine-rich G3K dendrimer coating, which can interact with the MSC membrane and potentially engage cell surface receptors to trigger intracellular signaling cascades. We also cannot exclude the possibility that some of the observed transcriptional changes may partially reflect a cellular response to the cationic nanogel coating; however, the functional validation experiments—enhanced migration toward CXCL12 (**Figure 4G**) and the preserved immunomodulatory gene expression even under cfDNA challenge (**Figure 4F**)—indicate that these changes are functionally meaningful rather than merely stress artifacts. Taken together, these findings demonstrate that MSC-G3K@T serves as an effective therapeutic agent for targeted immunomodulation.

### MSC-G3K@T synergistically suppresses CD4^+^ T cell activation and promotes an immunosuppressive phenotype

To verify the *in vitro* immunosuppressive and immunomodulatory properties of MSC-G3K@T, we conducted a coculture assay in which mouse CD4^+^ T cells were cocultured with PBS, G3K nanogel, MSCs, tacrolimus, MSC-G3K, and MSC-G3K@T for 5 days (**Figure 5A**). MSC-G3K@T was the most effective at suppressing CD4^+^ T-cell proliferation, outperforming all control groups—including free tacrolimus, MSC-G3K, and unmodified MSCs (**Figure 5B and G**). This enhanced suppressive effect was linked to a shift in T-cell lineage commitment. Flow cytometry showed that MSC-G3K@T treatment expanded the regulatory T cell population (Tregs, CD25^+^FOXP3^+^, **Figure 5C-D**) while reducing pro-inflammatory T-helper 17 cells (T_H_17, CD4^+^IL-17A^+^, **Figure 5E-F**). As a result, the Treg/T_H_17 ratio rose substantially, pointing to a restoration of immune balance (**Figure 5H**). The MSC-G3K group (without tacrolimus) also showed clear immunosuppressive activity, promoting Treg induction and suppressing T_H_17 responses. This likely reflects the enhanced immunomodulatory capacity of the nanoengineered MSCs: RNA-seq and qPCR analyses confirmed that MSC-G3K upregulates *TGF-β*, *IL-10*, and *PTGES*, all of which are implicated in driving Treg differentiation while limiting T_H_17 polarization (**Figure 4D-F**).

We next asked whether the enhanced immunoregulatory signature of MSC-G3K@T, identified by RNA-seq (**Figure 4**), would be reflected in the transcriptional response of cocultured CD4^+^ T cells. Among the T-cell fate regulators examined by qPCR, *RORγt*—a transcription factor that drives T_H_17 differentiation—was markedly downregulated in the MSC-G3K@T group (**Figure 5I**). As *RORγt* is essential for T_H_17 differentiation, its downregulation likely accounts for both the reduced T_H_17 population and the marked drop in IL-17A secretion (**Figure 5J**). *FOXP3*, the transcription factor that drives Treg development, was significantly upregulated, consistent with the expansion of the Treg population described above (**Figure 5K**). We also detected higher expression of IL-10, an anti-inflammatory cytokine released by Tregs, further supporting this immunosuppressive shift (**Figure 5L**).

Together, the data point to a synergistic mechanism: the immunosuppressive activity of MSC-G3K@T exceeds what would be expected from its individual components alone. The platform effectively rewires the transcriptional network in CD4^+^ T cells, simultaneously suppressing the pro-inflammatory T_H_17 program (via *RORγt*/IL-17) and promoting the anti-inflammatory Treg program (via* FOXP3*/IL-10). This multi-pronged mechanism, which is rooted in the transcriptomically-potentiated state of the engineered MSCs and directly corrects the Treg/T_H_17 imbalance implicated in LN pathogenesis, underscores the platform’s superior capability to restore immune homeostasis* in vitro*.

### *In vivo* targeting and responsive drug release of MSC-G3K@T

Having established the potent *in vitro* cfDNA binding and immunomodulatory capabilities of MSC-G3K@T, we next sought to validate its targeted delivery and on-demand drug release profile *in vivo*. For MSC survival *in vivo*, we have performed a subcutaneous implantation experiment in immunocompetent BALB/c mice comparing DiD-labeled MSC-G3K@T and native MSCs. *In vivo* fluorescence imaging over 7 days demonstrated that MSC-G3K@T exhibited significantly stronger and more persistent fluorescent signal compared to native MSCs, which rapidly declined within 3 days (**Figure S15**). This result indicates that the G3K nanogel corona can partially protect the engineered MSCs from host immune clearance, thereby prolonging their *in vivo* residence.

Having confirmed improved stability, we hypothesized that the platform would leverage the dual-homing mechanism—MSC’s innate tropism and G3K’s affinity for renal cfDNA—for precise accumulation in inflamed kidneys. To track the system *in vivo*, MSCs and the loaded tacrolimus were labeled with DiD and Cy5 fluorescent dyes, respectively. In MRL/lpr mice, MSC-G3K@T showed stronger and more sustained kidney accumulation than free tacrolimus or native MSCs. This pattern held across all imaging time points, from 2 to 72 hours after injection (**Figure 6A-B**). Quantitative biodistribution analysis confirmed this, showing that the renal accumulation of MSC-G3K@T was 20-fold and 2.2-fold greater than that of free tacrolimus and native MSCs, respectively, peaking at 24 hours (**Figure 6C-D**). Examination of major organ sections further confirmed the renal-specific targeting of MSC-G3K@T, with intense fluorescence localized to the kidneys and minimal signals detected in the heart, liver, spleen, and lungs (**Figure 6E and Figure S16**). This demonstrates that the engineering strategy successfully overcomes the poor targeting and rapid clearance that limit conventional MSC and drug therapies.

To corroborate these imaging-based findings with direct drug quantification, we measured tacrolimus concentrations in plasma and kidney tissues by LC-MS/MS at multiple time points post-injection. MSC-G3K@T significantly prolonged renal drug retention and increased renal drug exposure compared to an equivalent dose of free tacrolimus (**Figure S17**), confirming that MSC-mediated delivery enhances both systemic circulation time and kidney-selective drug accumulation.

To verify that this pronounced renal accumulation is driven specifically by the pathological microenvironment rather than by passive physical entrapment, we performed biodistribution studies in healthy MRL/MpJ mice—the genetically matched, non-lupus background strain. In healthy mice, little renal accumulation of MSC-G3K@T was observed (**Figure S18**). This difference suggests that the kidney homing seen in lupus mice depends on features of the disease microenvironment—in particular, the abundance of anionic cfDNA, which can retain the cationic G3K nanogel through electrostatic interactions.

We next tracked the metabolic fate of the nanogel component. FITC-labeled G3K nanogels were monitored over 7 days after MSC-G3K@T injection in MRL/lpr mice—a timeframe that corresponds to our weekly dosing schedule. Renal fluorescence declined steadily, reaching near-background levels by day 7, with no signs of long-term tissue retention (**Figure S19**). These results suggest that nanogel fragments are cleared within a single treatment cycle, which would help avoid cumulative renal exposure.

To examine targeting at the cellular level, we imaged kidney sections from MRL/lpr mice. Confocal microscopy from mice injected with DiD-labeled MSC-G3K@T revealed extensive colocalization (white fluorescence) with both iNOS positive M1 and CD4^+^ T cells, indicating successful homing to the key immune cell populations driving LN pathology (**Figure 6F**). Little colocalization was seen in control groups. This provides direct visual evidence that the dual-targeting strategy enables MSC-G3K@T to efficiently navigate to and engage with inflammatory lesions.

To rigorously validate that drug release is triggered by the pathological oxidative environment, we employed a triple-labeling strategy: MSCs (CellMask orange, red), G3KDSP nanogels (FITC, green), and tacrolimus (Cy5, yellow). In MRL/lpr kidneys, clear spatial separation of the tacrolimus and nanogel signals from the MSC carrier was observed at 12- and 24-hour post-injection (**Figure 6G**). This dispersion is indicative of the ROS-triggered disassembly of the nanogel, accompanied by the liberation of its loaded therapeutic payload. Such a process is crucial for achieving high local therapeutic concentrations at the disease site. Crucially, when the identical triple-labeled MSC-G3K@T was administered to healthy MRL/MpJ mice—which lack the renal oxidative microenvironment—the three fluorescence signals remained tightly co-localized with the MSC membrane, with negligible spatial separation (**Figure S20**).

Collectively, these* in vivo* findings corroborate our design principle: MSC-G3K@T functions as a sophisticated targeted delivery system that (1) utilizes dual-homing for enhanced renal accumulation, (2) actively engages with pathogenic immune cells, and (3) undergoes pathological stimulus-responsive drug release within the inflamed tissue (**Figure 6H**).

### MSC-G3K@T ameliorates disease progression in MRL/lpr mice

The *in vivo* therapeutic capacity of MSC-G3K@T was further evaluated in the lupus-prone MRL/lpr mouse strain, which develops spontaneous disease. Mice received weekly intravenous administrations of saline (control), G3KDSP nanogels, MSCs, tacrolimus, MSC-G3K, or MSC-G3K@T over four weeks (**Figure 7A**). The MSC-G3K@T treatment group exhibited the most pronounced amelioration of systemic lupus symptoms, including an obvious alleviation in the severity of facial skin injuries (**Figure 7B and Figure S21**) and a marked decrease in spleen and lymph node sizes (**Figure 7C and Figure S22**). All treated groups maintained stable body weight, suggesting the general safety of the regimens (**Figure 7D**).

A pivotal mechanism underlying this efficacy is the system’s ability to neutralize pathogenic cfDNA *in vivo*. MSC-G3K@T and MSC-G3K were significantly more effective at clearing cfDNA from the bloodstream and the kidney tissue than other treatments (**Figure 7E and Figure S23A**). This can be attributed to the synergistic effect of the prolonged circulation and enhanced renal retention of the engineered MSCs, combined with the high nucleic acid binding affinity of the G3KDSP nanogel corona. In contrast, free G3KDSP nanogels, despite their intrinsic cfDNA affinity, showed only a modest reduction in systemic cfDNA, likely because the nanogel-cfDNA complexes themselves might be partially cleared or internalized by renal cells.

We next asked whether cfDNA clearance translates into suppressed NF-κB signaling in the kidney. Immunofluorescence staining showed that all G3K-containing formulations significantly lowered NF-κB p65 levels relative to saline, whereas naïve MSCs did not (**Figure S23B-C**). A likely explanation is that the cationic nanogel binds and neutralizes cfDNA through electrostatic interactions, preventing it from engaging TLR9. This would reduce MyD88-dependent signaling and, in turn, limit NF-κB p65 nuclear translocation. The data thus demonstrate that scavenging of cfDNA by the nanogel suppresses the TLR9–NF-κB axis *in vivo* and highlights the MSC carrier as an essential component for durable cfDNA clearance.

Renal dysfunction markers were substantially improved by MSC-G3K@T. Anti-dsDNA antibody titers, a defining serological feature of SLE, were effectively reduced (**Figure 7F**). MSC-G3K@T also led all groups in restoring glomerular filtration, with the largest decreases in proteinuria, serum creatinine, and BUN (**Figure 7G-I**). Renal TNF-α and IL-6 were downregulated in parallel (**Figure S24**), providing molecular correlates of this protection.

These functional benefits were mirrored at the tissue level. Hematoxylin and eosin (H&E) and Masson's trichrome staining demonstrated that MSC-G3K@T alleviated the principal histological features of LN—including glomerular inflammation, perivascular cuffing, and interstitial infiltration—more effectively than any other treatment (**Figure 8A-C**). Periodic acid-Schiff (PAS) staining pointed to reduced fibrosis (**Figure S25**). Immunofluorescence showed that glomerular deposition of IgG and C3, central to immune complex-mediated renal damage, was lowest in the MSC-G3K@T group (**Figure 8D-F**).

Together, these *in vivo* findings support a multi-pronged therapeutic mechanism. By scavenging cfDNA and reducing autoantibodies, MSC-G3K@T disrupts the autoimmune cycle that drives LN, while locally delivering immunosuppression and immunomodulation. This integrated action—combining cfDNA clearance, targeted drug release, and enhanced cellular therapy—alleviated both systemic and renal manifestations of lupus, reducing cellular inflammation and antibody-mediated damage in the kidney. These results position MSC-G3K@T as a promising strategy for LN and potentially other autoimmune disorders.

### MSC-G3K@T reverses systemic immune dysregulation in MRL/lpr mice

We next asked whether the renal protection observed in MSC-G3K@T-treated mice reflected a broader restoration of immune tolerance. Flow cytometric analysis of splenocytes and peripheral blood mononuclear cells (PBMCs) showed that MSC-G3K@T expanded Tregs (CD4^+^FOXP3^+^) (**Figure 9B-C and Figure S26A**) while reducing T_H_17 (CD4^+^IL-17A^+^) and T follicular helper (Tfh, CXCR5^+^PD-1^+^) cell populations (**Figure 9D-G and Figure S26B, C**). The resulting increase in the Treg/T_H_17 ratio (**Figure S27**) suggested a shift toward immune homeostasis. Treg expansion may have helped restrain T_H_17 and Tfh responses, thereby dampening the autoimmune cascade. MSC-G3K@T also influenced innate immunity: neutrophils (LY-6G^+^CD11B^+^) were decreased (**Figure 9H-I**), and M2 macrophage polarization (F4/80^+^CD206^+^) was promoted, shifting the M2/M1 balance toward an anti-inflammatory profile (**Figure 9J-K and Figure S26D**).

This systemic correction of immune dysregulation can be traced to three complementary actions. The G3KDSP nanogel scavenges cfDNA, reducing a potent trigger of innate immunity. Tacrolimus, released in a controlled manner, directly suppresses T-cell activation. At the same time, the nanoengineered MSCs exert enhanced immunomodulatory effects that help reshape the local immune environment.

Importantly, the therapeutic effects described above were achieved without notable off-target toxicity. MSC-G3K@T halted the progression of lupus nephritis while showing minimal systemic toxicity *in vivo* (**Figure S28**). Serum hepatic markers—ALP, ALT, and AST—remained within normal ranges in treated mice (**Figure S29**), consistent with a favorable safety profile. Together, these results indicate that MSC-G3K@T combines direct renal protection with systemic immune restoration, supporting its further evaluation as a safe therapeutic approach for LN and related autoimmune conditions.

We also recognize that the weekly intravenous administration schedule employed in this study may be impractical for the long-term management of a chronic relapsing disease such as lupus. Reducing the dosing frequency—for instance, to biweekly or monthly intervals—while maintaining therapeutic efficacy would be an important goal for future clinical translation, and alternative dosing regimens should be explored in subsequent studies.

## Conclusion

In summary, we have successfully engineered a nanoengineered MSC therapeutic, termed MSC-G3K@T, which integrates the dynamic drug-delivery and cfDNA-scavenging capabilities of a cationic dendritic nanogel with the innate immunomodulatory functions of MSCs. This platform represents a notable advancement in cell engineering by modulating the MSC phenotype, endowing it with a transcriptomic profile favorable for enhanced homing (upregulated *CXCR4*) and immunoregulation (upregulated *IL-10* and *TGF-β1*). Through a dual-targeting mechanism—leveraging both the MSC’s innate tropism and the nanogel’s affinity for pathogenic cfDNA—MSC-G3K@T achieves specific accumulation in inflamed kidneys. At the disease site, the platform acts through three complementary mechanisms: (1) The G3K nanogel corona scavenges cfDNA, reducing TLR9–NF-κB signaling. (2) Tacrolimus is released in response to local ROS, providing controlled immunosuppression. (3) The nanoengineered MSCs help correct the Treg/T_H_17 imbalance, suppress Tfh responses, and promote M2 macrophage polarization. In the MRL/lpr model, this combination achieved broad therapeutic effects: systemic autoimmunity was reduced, renal function improved, and glomerular structure was preserved. No significant toxicity was observed. These findings highlight MSC-G3K@T as a promising candidate for LN and suggest that similar cell-engineering strategies could be applied to other autoimmune disorders.

A practical challenge for clinical translation is the multi-step fabrication process, which could complicate large-scale production and lead to batch-to-batch variability. In the near term, these challenges may be partially addressed through automation of nanogel synthesis and MSC conjugation using microfluidic systems, implementation of stringent quality control parameters (particle size, zeta potential, drug loading, viable cell count), and development of lyophilized G3K@T nanogels as standardized reagents for on-demand MSC engineering. Looking forward, a more fundamental solution would be to explore structurally simpler cationic materials, as potential substitutes for the G3K dendrimer. Such substitution may substantially simplify the fabrication process and improve the clinical translatability of this platform.

## Supplementary Material

Supplementary figures and table.

## Figures and Tables

**Figure 1:**
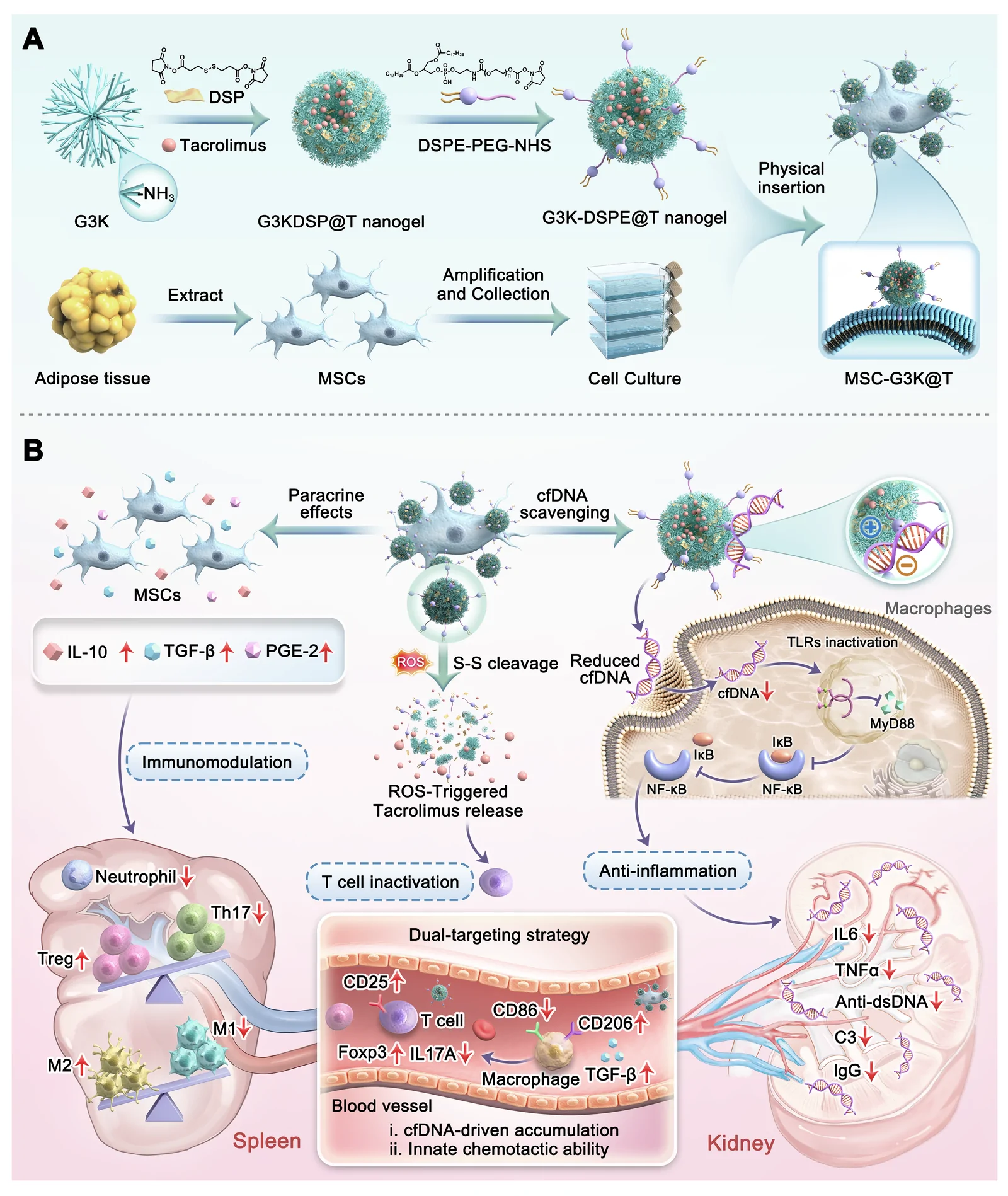
Schematic diagram of the nanoengineered MSC platform for synergistic treatment of LN. (A) Preparation process of MSC-G3K@T. (B) The platform executes synergistic therapy through (i) ROS-triggered tacrolimus release for T-cell suppression, (ii) MSC-mediated immunomodulation, and (iii) G3K-dependent cfDNA scavenging to block pro-inflammatory TLR/NF-κB activation.

**Figure 2:**
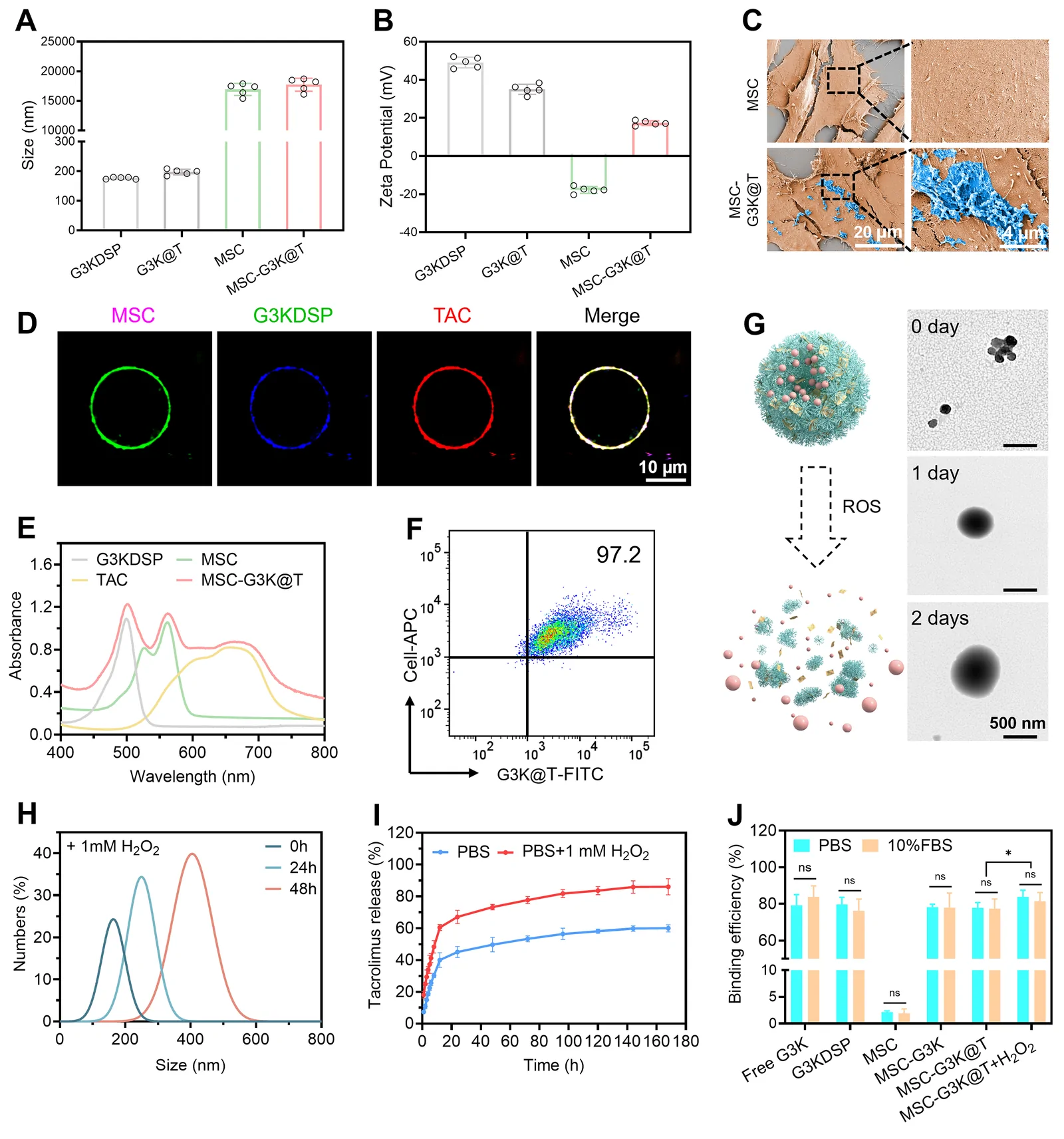
Fabrication, characterization, and functional validation of the nanoengineered MSC platform (MSC-G3K@T). (A) Hydrodynamic size and (B) Zeta potential of G3KDSP nanogels, drug-loaded G3K@T nanogels, naive MSCs, and the final MSC-G3K@T construct. (C) SEM images showing spherical G3K@T nanogels firmly attached to the MSC surface. (D) Confocal microscopy images confirmed co-localization of CellMask orange-labeled MSCs (violet), FITC-labeled G3KDSP nanogels (green), and Cy5-labeled tacrolimus (red), verifying the integrated hybrid structure. (E) Absorption spectra (UV-Vis) of the individual components and the MSC-G3K@T hybrid. (F) Flow cytometry analysis further confirmed successful construction, with cells appearing in the double-positive quadrant for CellMask Orange and FITC. (G) TEM images of G3K@T nanogels after 7-day incubation with 1 mM H₂O₂; the disintegration observed here indicates ROS-responsive behavior. (H) Shift in hydrodynamic size of nanogels after 72 hours of H₂O₂ exposure, consistent with disassembly. (I) *In vitro* tacrolimus release profile from MSC-G3K@T, demonstrating sustained release in physiological PBS (pH 7.4) and significantly boosted release under a pathological oxidative environment (1 μM H₂O₂). (J) Evaluation of DNA binding capacity in PBS and 10% FBS, confirming the potent and serum-resistant cfDNA scavenging capability of the G3K component and MSC-G3K@T, in contrast to naive MSCs. Data are presented as mean ± SD (n ≥ 3). Statistical significance was determined by one-way ANOVA with Tukey’s post hoc test; *p < 0.05, ns, not significant.

**Figure 3:**
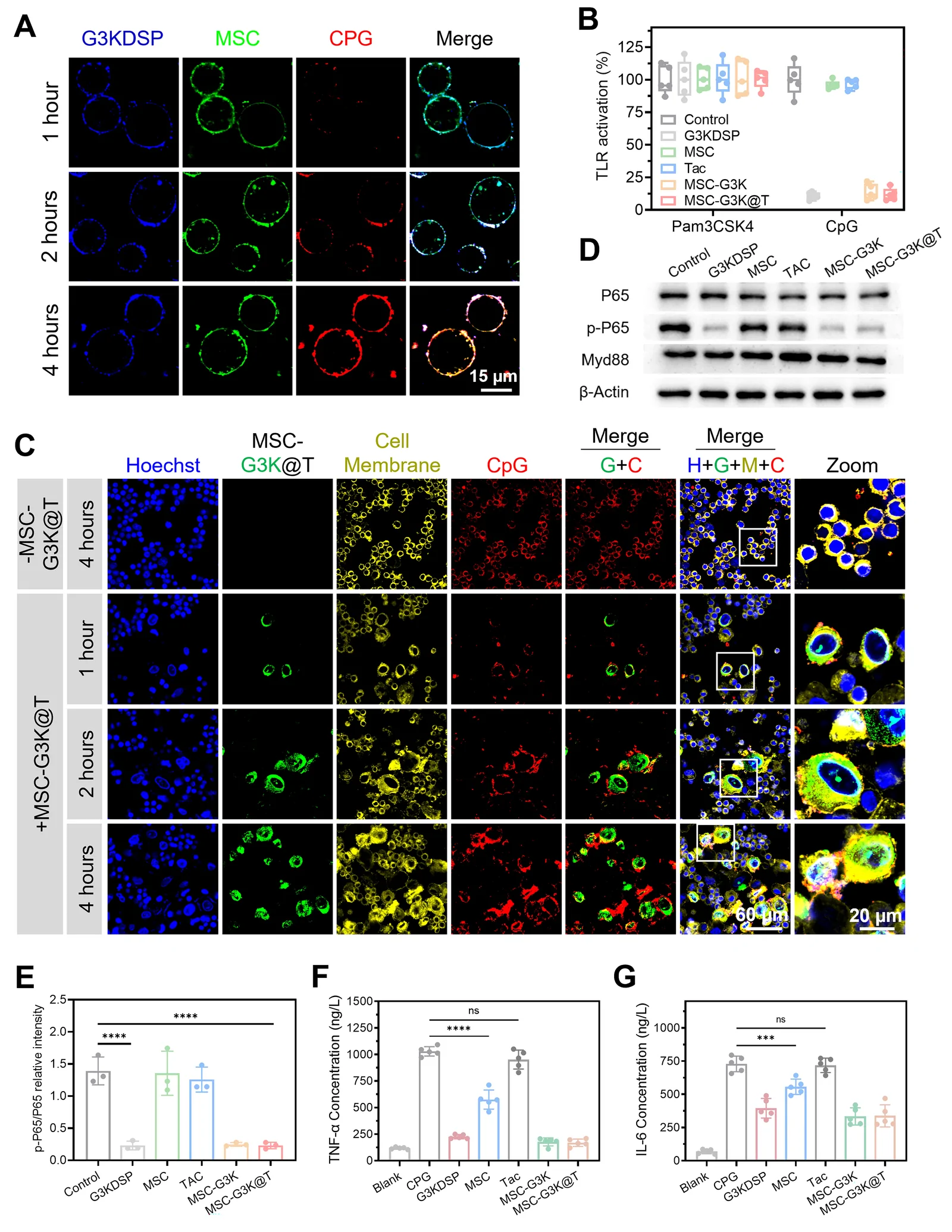
Extracellular cfDNA sequestration by MSC-G3K@T inhibits TLR9-mediated inflammatory response. (A) Confocal images showing the time-dependent binding of Quasar 670-labeled CpG-ODN2006 (red) to the membrane of MSC-G3K@T (Blue: FITC-labeled G3KDSP nanogels; Green: CellMask orange-labeled MSC membrane). (B) Suppression of CpG-induced TLR9 signaling within Ramos Blue™ reporter cells treated with G3KDSP nanogels, MSC-G3K, and MSC-G3K@T. TLR2 activation by Pam3CSK4 served as a negative control. (C) Competitive cfDNA uptake assay. Top row: RAW264.7 macrophages cultured solely with Quasar 670-tagged CpG for 4 hours exhibited robust intracellular uptake. Bottom rows: Co-incubation of CpG with both MSC-G3K@T and RAW264.7 cells over 1, 2, and 4 hours. CpG is progressively captured by the MSC-G3K@T surface, drastically reducing its uptake by RAW264.7 cells. RAW264.7 cells and MSCs are distinguished by size: 10 μm for RAW264.7 cells; 20 μm for MSCs. Blue: Hoechst-stained cell nuclei. Green: FITC-labeled G3KDSP nanogels; Yellow: CellMask orange-labeled cell membrane; Red: Quasar 670-labeled CpG-ODN2006. (D) Immunoblotting assessment of NF-κB p65, phospho-p65 (p-p65), and MyD88 in CpG-stimulated RAW264.7 cells following treatment with G3KDSP nanogels, MSC, tacrolimus, MSC-G3K, or MSC-G3K@T. (E) Quantitative analysis of p-p65/p65 relative intensity from (D). (F, G) Subsequent suppression of proinflammatory cytokine (D) TNF-α and (E) IL-6 production in RAW264.7 cells. Data are expressed as mean ± SD (n = 5). Statistical significance was assessed using one-way ANOVA with Tukey's post hoc test; ***p < 0.001, ****p < 0.0001, ns, not significant.

**Figure 4:**
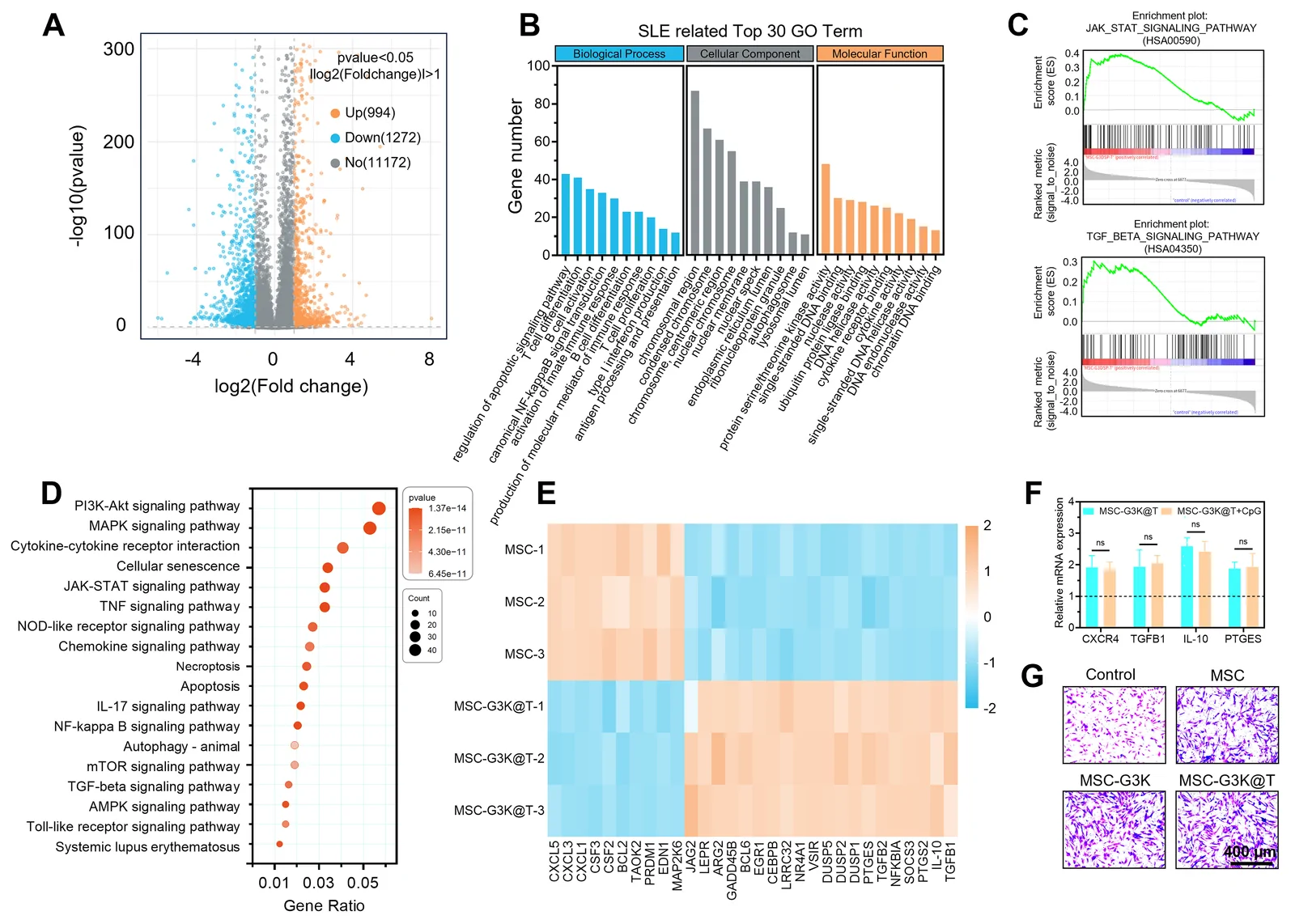
Nano-engineering potentiates MSC homing and immunomodulatory function. (A) Volcano plot of DEGs between MSC-G3K@T and naïve MSCs. (B) GO enrichment analysis of DEGs covering BP, CC, and MF, showing terms related to immune regulation. (C) GSEA plots demonstrating significant enrichment of key pathways in MSC-G3K@T: JAK-STAT and TGF-β signaling. (D) KEGG enrichment analysis. (E) Heatmap for key immunomodulatory genes and homing-related genes, showing consistent upregulation in MSC-G3K@T across replicates. (F) qPCR validation of key upregulated immune-related genes in MSC-G3K@T compared to naïve MSCs. (G) Transwell migration assay toward CXCL12, demonstrating significantly enhanced chemotactic ability of MSC-G3K@T compared to naïve MSCs. Data were presented as mean ± SD (n = 3). Statistical significance was determined in comparison to the naïve MSCs group, calculated by one-way ANOVA followed by post hoc tests, ns, not significant.

**Figure 5:**
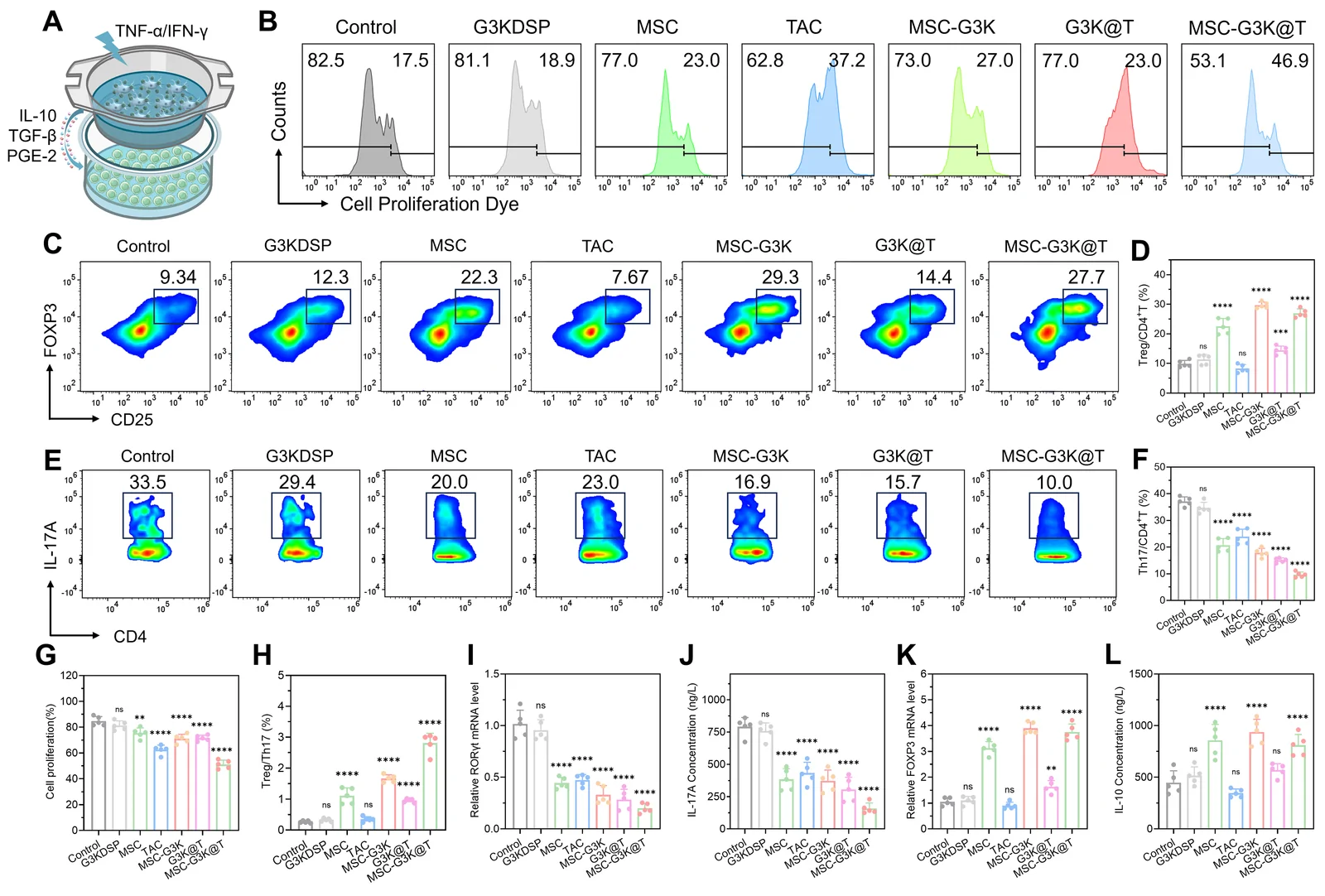
MSC-G3K@T potently suppresses CD4^+^ T cell expansion and promotes an immunosuppressive differentiation profile via transcriptional regulation. (A) Graphical overview of the T cell co-culture experiment. (B) Proliferation of CD4⁺ T cells measured by flow cytometry. (C-D) CD25^+^FOXP3^+^ Treg cell populations, with representative plots and pooled quantification. (E, F) CD4^+^IL-17A^+^ T_H_17 cell populations, with representative plots and pooled quantification. (G) Suppression rate of CD4⁺ T cell proliferation. (H) Calculated Treg/T_H_17 ratio. (I-L) Transcript levels of RORγt (I) and FOXP3 (K) by qPCR, alongside secreted IL-17A (J) and IL-10 (L) quantified by ELISA. All data are mean ± SD (n = 5). Statistical significance was determined by one-way ANOVA with Tukey's post hoc test; **p < 0.01, ***p < 0.001, ****p < 0.0001, ns, not significant.

**Figure 6:**
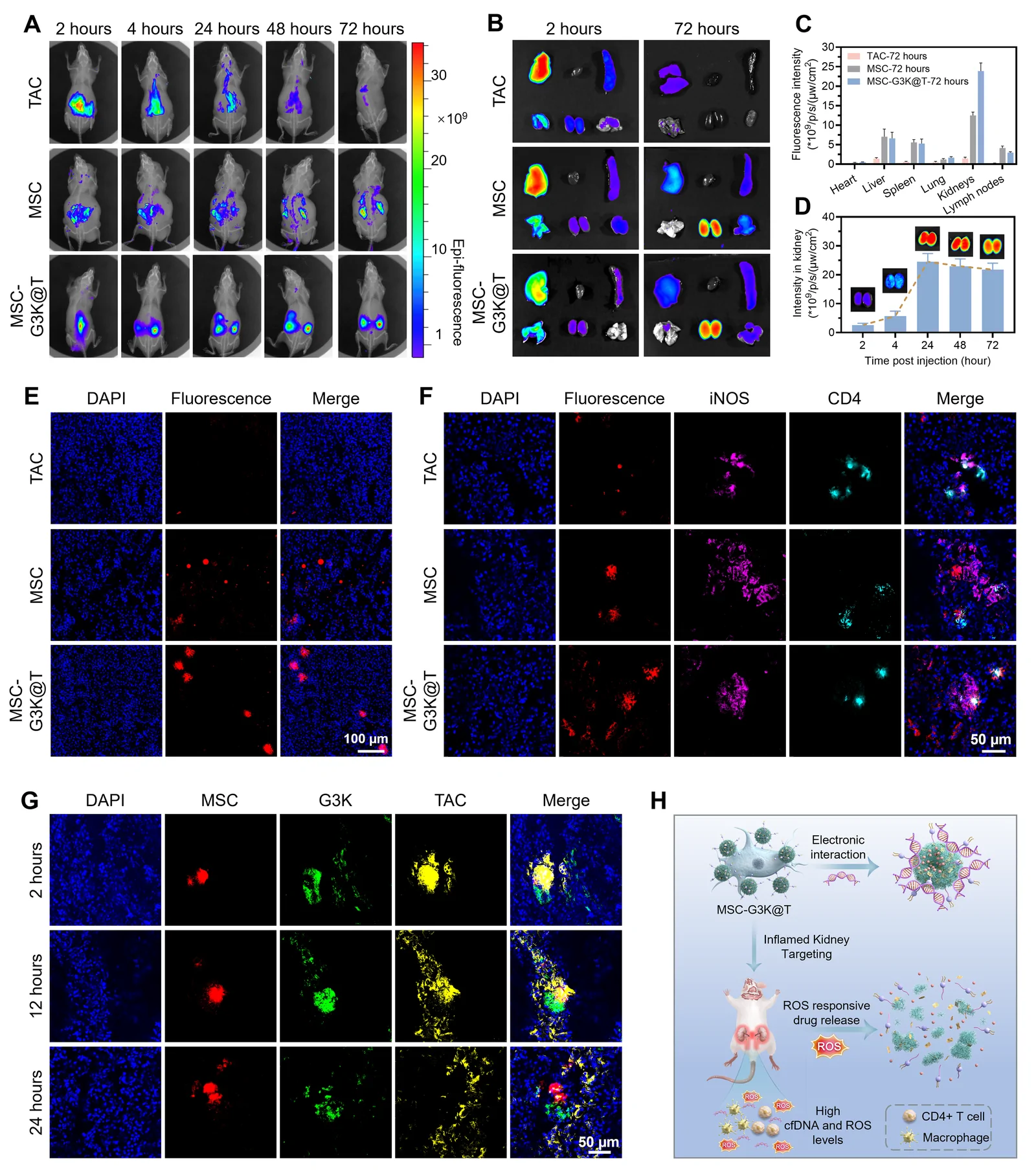
*In vivo* dual-targeting, inflammatory cell engagement, and ROS-responsive drug release of MSC-G3K@T in MRL/lpr mice. (A, B) Whole-body fluorescence imaging and (C, D) quantification of tissue distribution shown for DiD-labeled MSC-G3K@T, DiD-labeled MSCs and Cy5-labeled tacrolimus following intravenous administration of MSC-G3K@T, free MSCs, or free tacrolimus. (E) Representative fluorescence images of kidney sections from treated mice. (F) Confocal images showing colocalization (white spots) of Cy5-labeled tacrolimus (red), DiD-labeled MSC (red), and DiD-labeled MSC-G3K@T (red) with iNOS positive M1 macrophages (violet) or CD4^+^ T cells (green) in kidneys. (G) *In situ* visualization of ROS-responsive drug release: triple-labeled MSC-G3K@T (MSCs: CellMask orange, red; G3KDSP nanogels: FITC, green; tacrolimus: Cy5, yellow) shows spatial separation of drug and nanogel from the cell carrier in kidneys. (H) Schematic illustration of the proposed *in vivo* mechanism. Data are presented as mean ± SD (n = 3).

**Figure 7:**
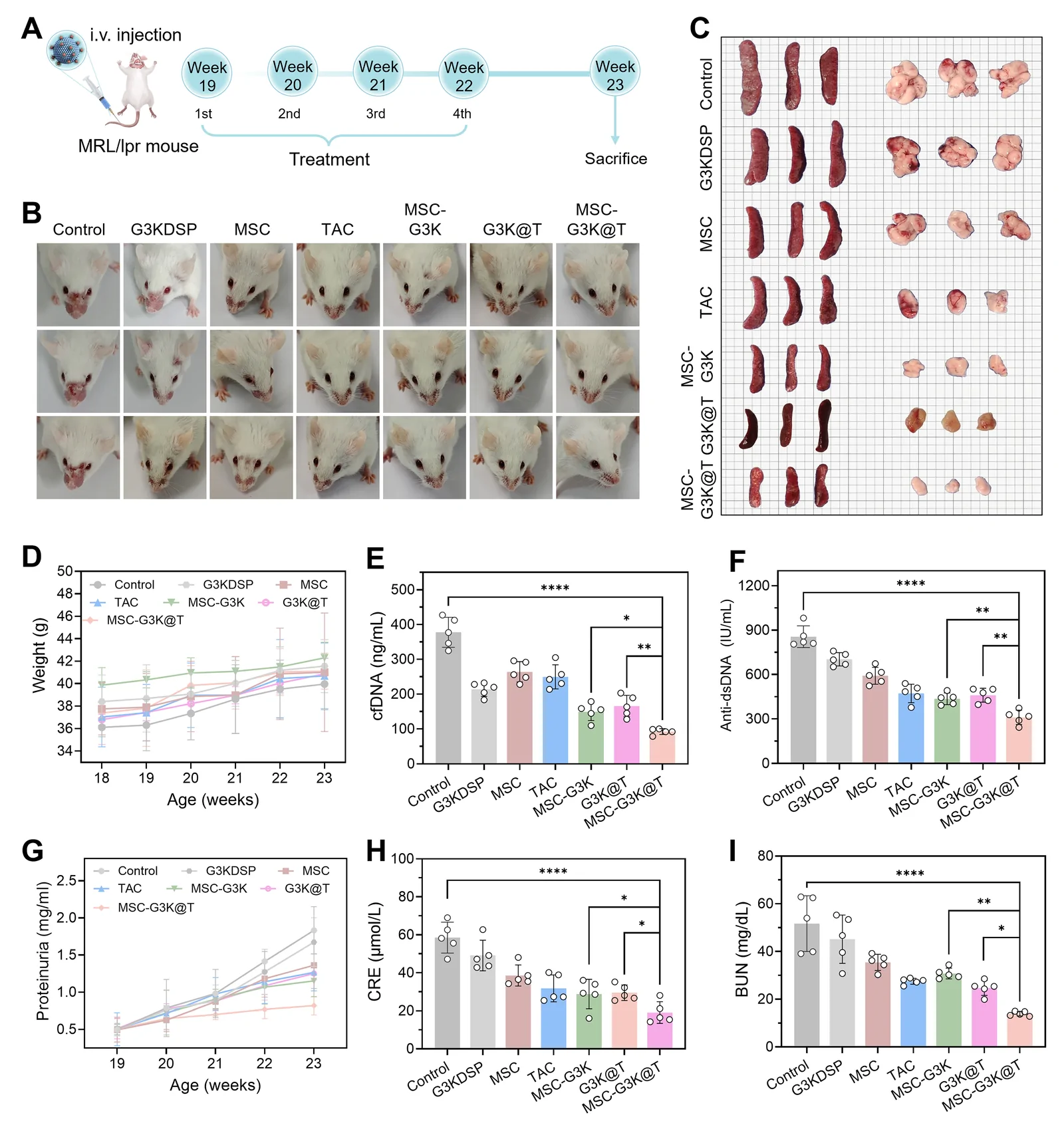
MSC-G3K@T treatment alleviates systemic lupus manifestations and restores renal function in MRL/lpr mice. (A) Therapeutic schedule. (B) Typical photographs of facial lesion area. (C) Spleen and lymph nodes size. (D) Body weight changes. (E) Plasma cfDNA levels. (F) Serum anti-dsDNA antibody levels. (G-I) Renal function markers: (G) proteinuria, (H) serum creatinine, and (I) blood urea nitrogen (BUN). Data are presented as mean ± SD (n = 5). Statistical significance was determined by one-way ANOVA with Tukey's post hoc test; *p < 0.05, **p < 0.01, ****p < 0.0001.

**Figure 8:**
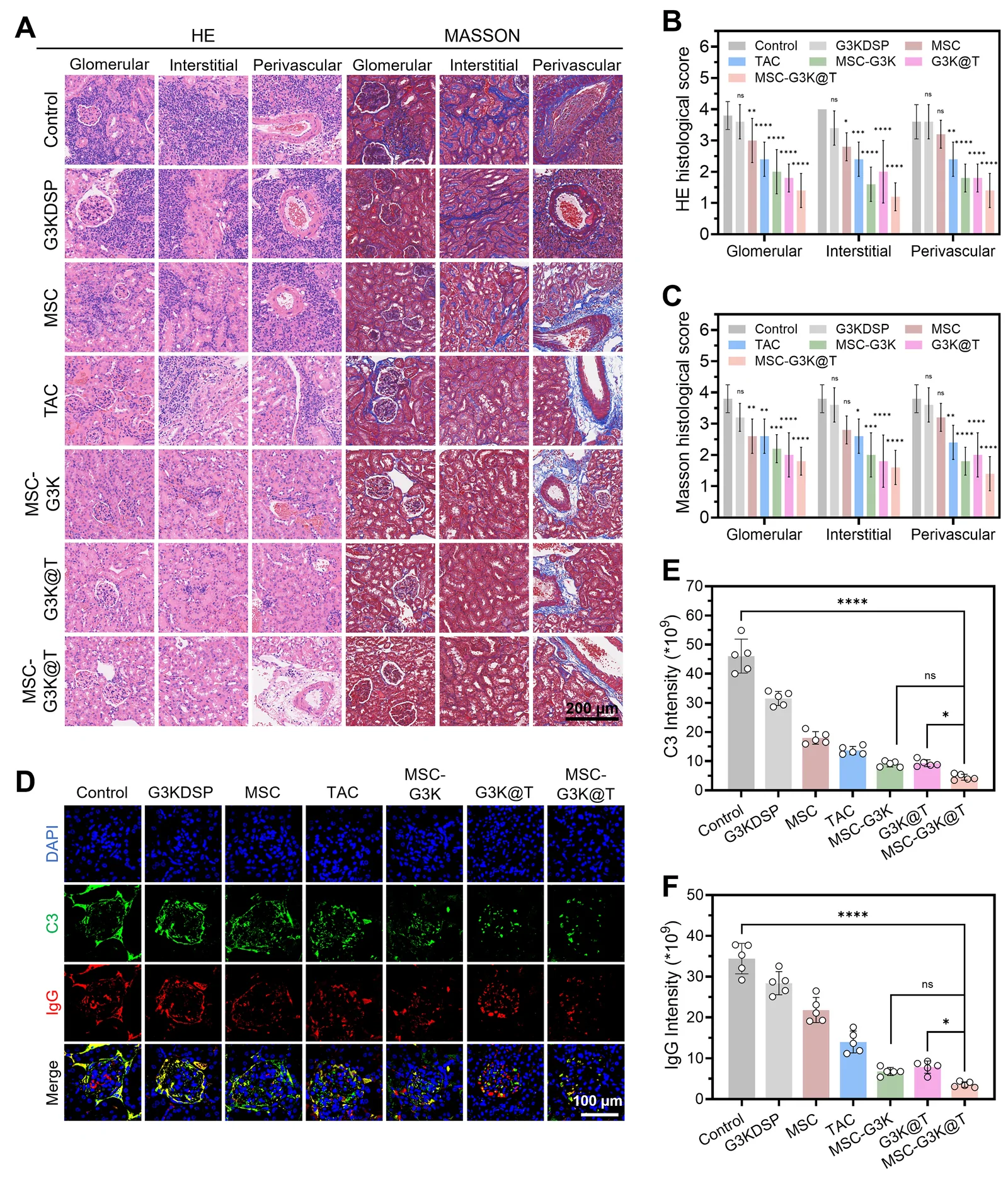
MSC-G3K@T ameliorates renal histopathology and accumulation of immune complexes in MRL/lpr mice. (A) Representative H&E and Masson-stained kidney sections. (B, C) Quantification of glomerular, interstitial, and perivascular inflammation based on HE and Masson’s trichrome. (D) glomerular C3 (green) and IgG (red) immunofluorescence. (E, F) Quantification of (E) C3 and (F) IgG fluorescence intensity in glomeruli. Data are presented as mean ± SD (n = 5). Statistical significance was determined by one-way ANOVA with Tukey's post hoc test; *p < 0.05, **p < 0.01, ***p < 0.001, ****p < 0.0001, ns, not significant.

**Figure 9:**
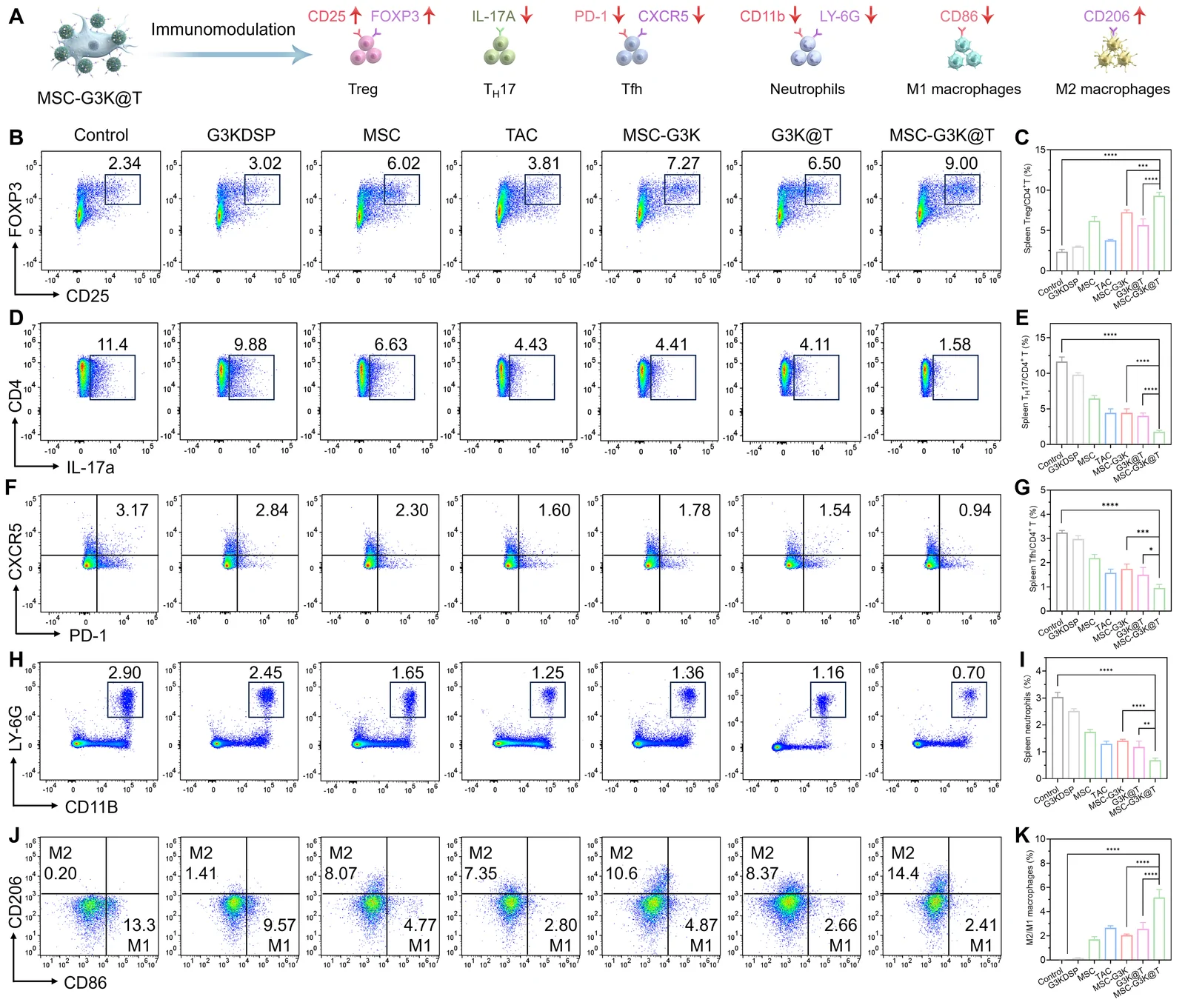
MSC-G3K@T restores immune homeostasis by reprogramming splenic immune cell populations in MRL/lpr mice. (A) Scheme of multi-mechanistic action of MSC-G3K@T in alleviating LN by modulating T cell subsets and inflammatory cells. (B, C) Flow cytometric of Treg cells (CD4^+^FOXP3^+^) in (B) representative plots and (C) quantitative summary. (D, E) Frequency of T_H_17 cells (CD4^+^IL-17A^+^) shown in (D) representative plots and (E) quantitative data. (F, G) Frequency of Tfh cells (CXCR5^+^PD-1^+^) shown in (F) representative plots and (G) quantitative data. (H, I) Analysis of pro-inflammatory neutrophils (LY-6G^+^CD11B^+^) in (H) representative plots and (I) quantitative summary. (J, K) Analysis of M2 macrophage polarization (F4/80^+^CD206^+^) and the M2/M1 ratio in (J) representative plots and (K) quantitative data. All flow cytometry data are from splenocytes. Data are presented as mean ± SD (n = 5). Statistical significance was determined by one-way ANOVA with Tukey's post hoc test; *p < 0.05, **p < 0.01, ***p < 0.001, ****p < 0.0001.

## Data Availability

The datasets used and/or analyzed during the current study are available from the corresponding authors upon reasonable request.

## Abbreviations

SLE: systemic lupus erythematosus
LN: lupus nephritis
MSC: mesenchymal stromal cells
cfDNA: cell-free DNA
ROS: reactive oxygen species
POSS: polyhedral oligomeric silsesquioxane
G3K: generation 3 poly(L-lysine) dendrimer
DSP: 3,3’-dithiodipropionic acid-di(N-succinimidyl ester)
TAC: tacrolimus
RNA-seq: RNA sequencing
qPCR: quantitative real-time polymerase chain reaction
TLR9: Toll-like receptor 9
MyD88: myeloid differentiation primary response 88
NF-κB: nuclear factor κB
TGF-β: transforming growth factor β
dsDNA: double-stranded DNA
TNF-α: tumor necrosis factor α
IL-6: interleukin 6
IL-10: interleukin 10
PBMC: peripheral blood mononuclear cells
Treg: regulatory T cells
T_H_17: T helper 17 cells
Tfh: T follicular helper cells
