# Supplementary material for: A nanoengineered MSC therapeutic for synergistic therapy of lupus nephritis via cfDNA clearance and targeted immunomodulation
Source: Theranostics. 2026 Sep 3;16(16):9145–64. doi: 10.7150/thno.136356 (PMC13684394; doi:10.7150/thno.136356)
Supplement: Supplementary file 1 [file thnov16p9145s1.pdf]

Supplementary

A Nanoengineered MSC Therapeutic for Synergistic Therapy of Lupus Nephritis via cfDNA Clearance and Targeted Immunomodulation

Jiang Tian[a,1], Qinyao Xu[b,1], Zexin Wang[b], Zeyu Wang[b], Haofang Zhu[b,*], and Lingyun Sun[a,c,*], Zhongwei Gu[d]

[a] Department of Rheumatology and Immunology, Nanjing Drum Tower Hospital, Affiliated Hospital of Medical School, Nanjing University, Nanjing, China

[b] Department of Rheumatology and Immunology, The First Affiliated Hospital of Anhui Medical University, Hefei, China

[c] Department of Rheumatology and Immunology, The Second Affiliated Hospital of Anhui Medical University, Hefei, China

[d] Research Institute for Biomaterials, Tech Institute for Advanced Materials, College of Materials Science and Engineering, Suqian Advanced Materials Industry Technology Innovation Center, Nanjing Tech University, Nanjing 211816, China

[1]: These authors contributed equally to this work.

*: Corresponding authors.

Email: hfzhu@ahmu.edu.cn, lingyunsun@nju.edu.cn.

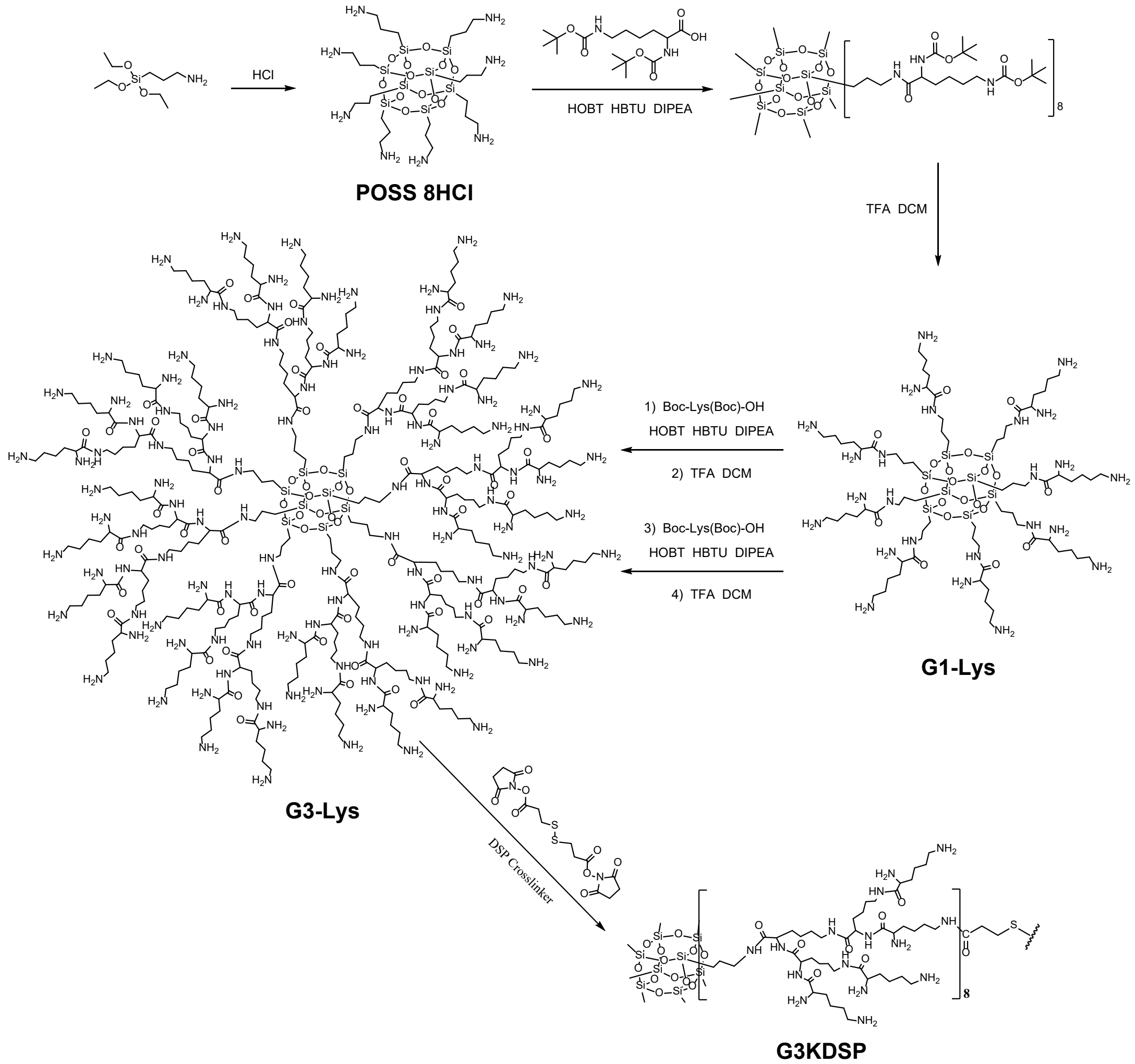

Figure S1. Synthesis route of the POSS-cored G3K dendrimer and G3KDSP nanogels.

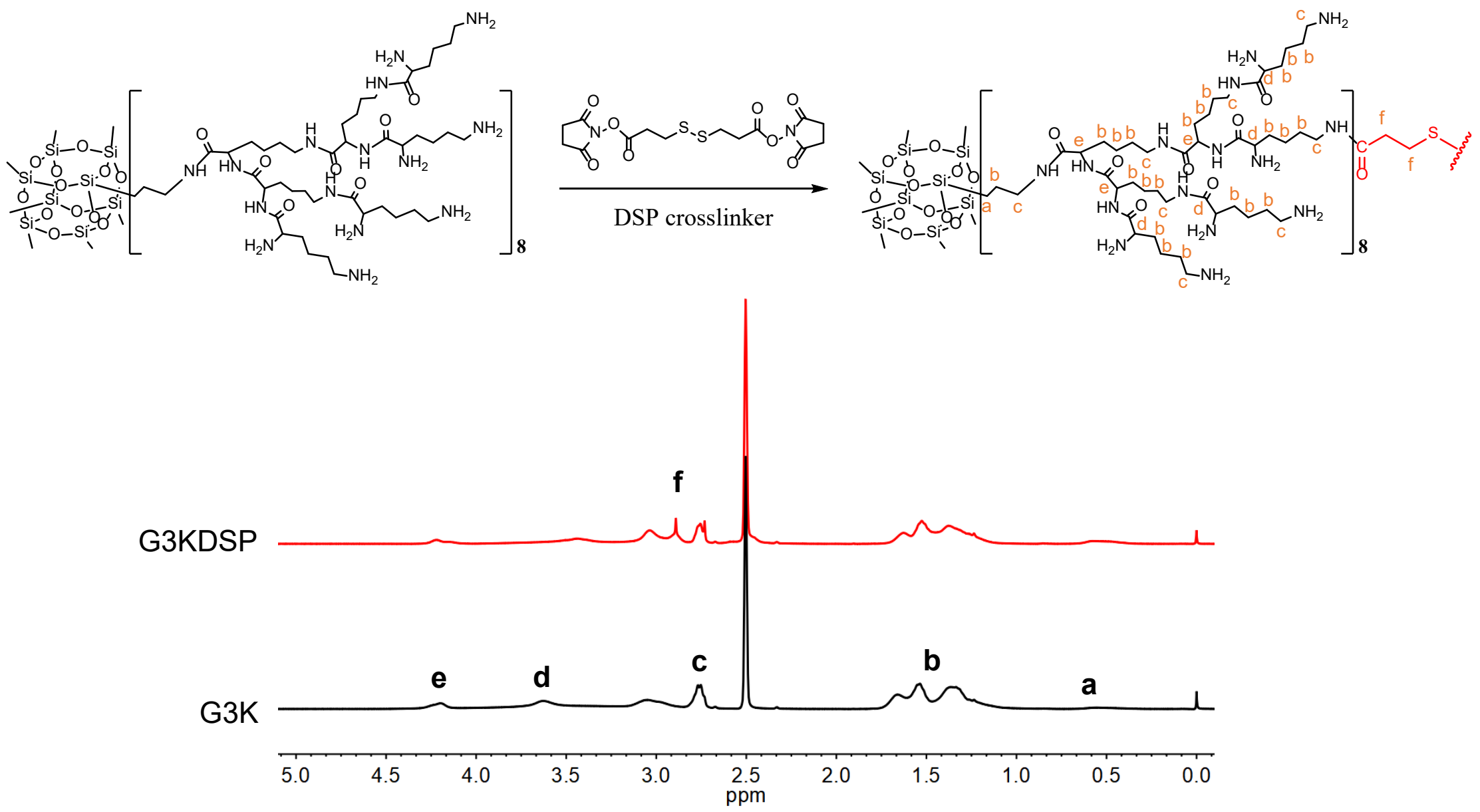

Figure S2. ^{1}H NMR spectrum of G3K dendrimer and G3KDSP nanogels.

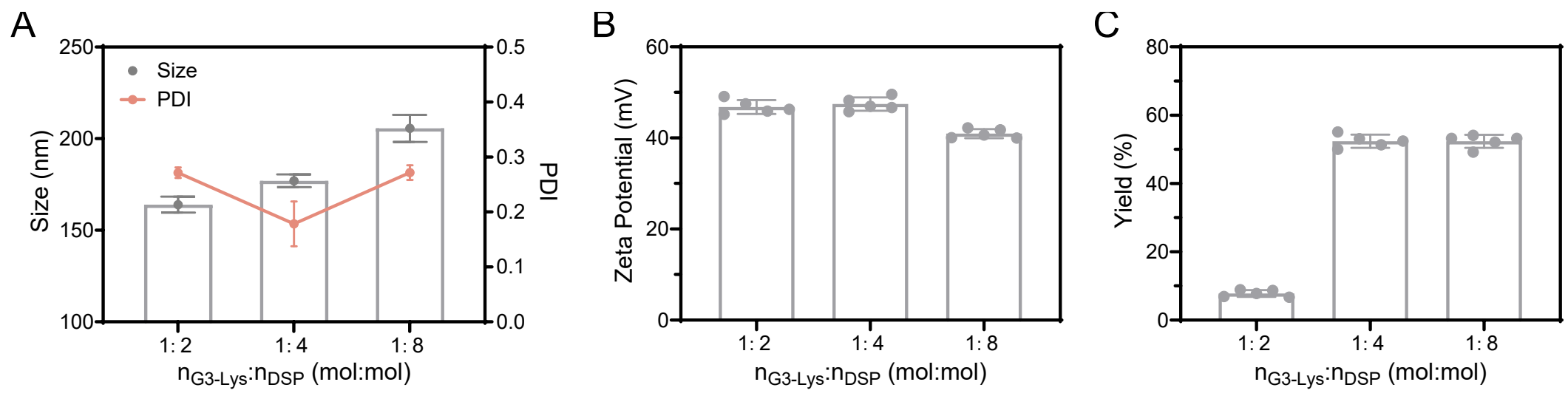

Figure S3. Evaluation of nanogel properties at different molar ratios of G3K to DSP crosslinker (1:2, 1:4, 1:8): (A) Hydrodynamic diameter measured by DLS, (B) Zeta potential, and (C) Reaction yield. The 1:4 ratio was selected as optimal based on the balanced performance across all three parameters. Data are presented as mean ± SD (n = 5).

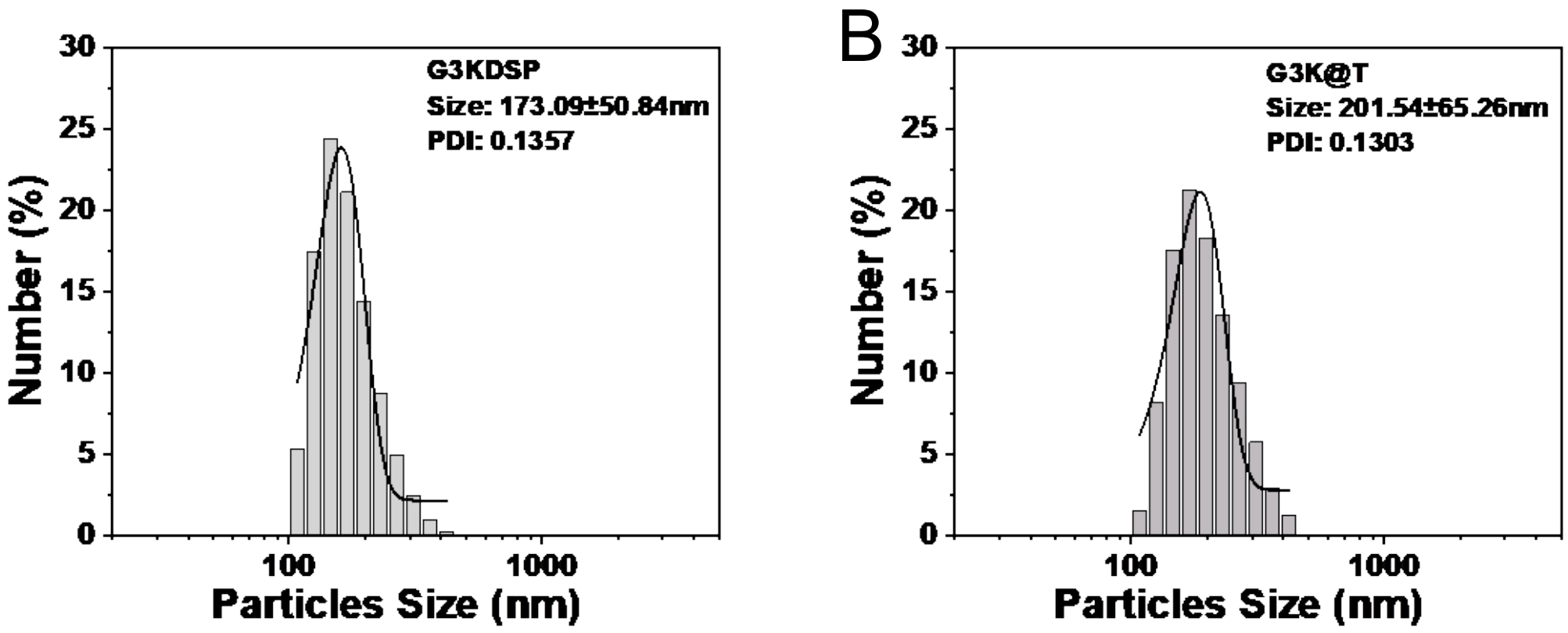

Figure S4. Hydrodynamic size distribution of G3KDSP and G3K@T nanogels measured by DLS, showing a slight increase upon drug loading.

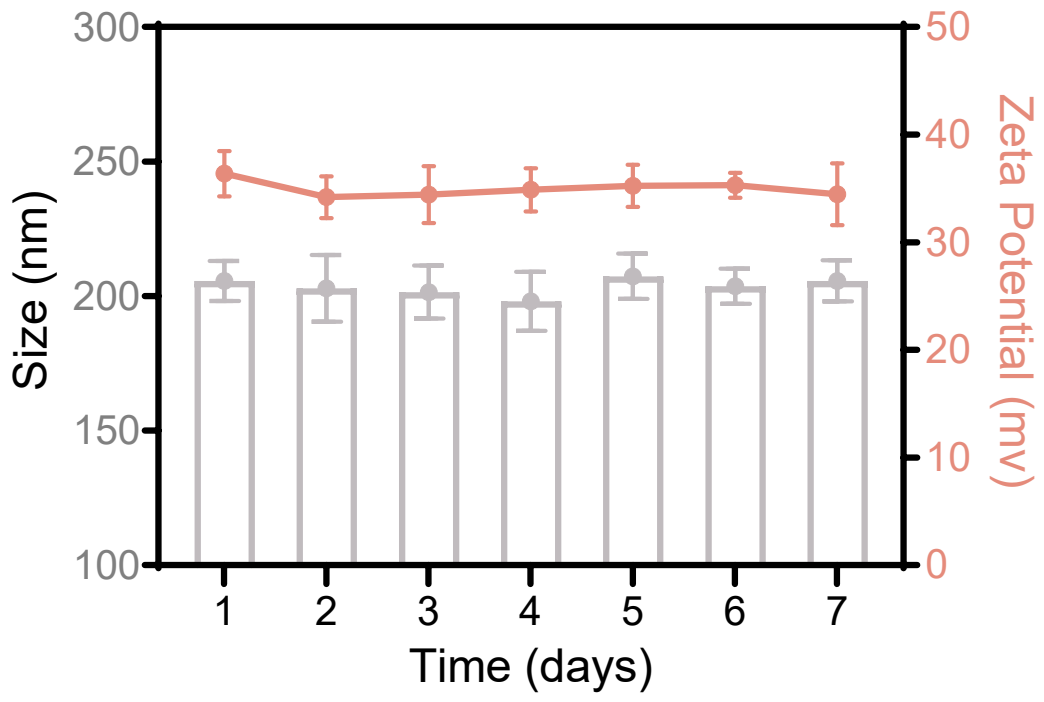

Figure S5. G3K@T nanogels maintained stable size and zeta potential over 7 days in PBS (pH 7.4) at 37 °C. Data are presented as mean ± SD (n = 3).

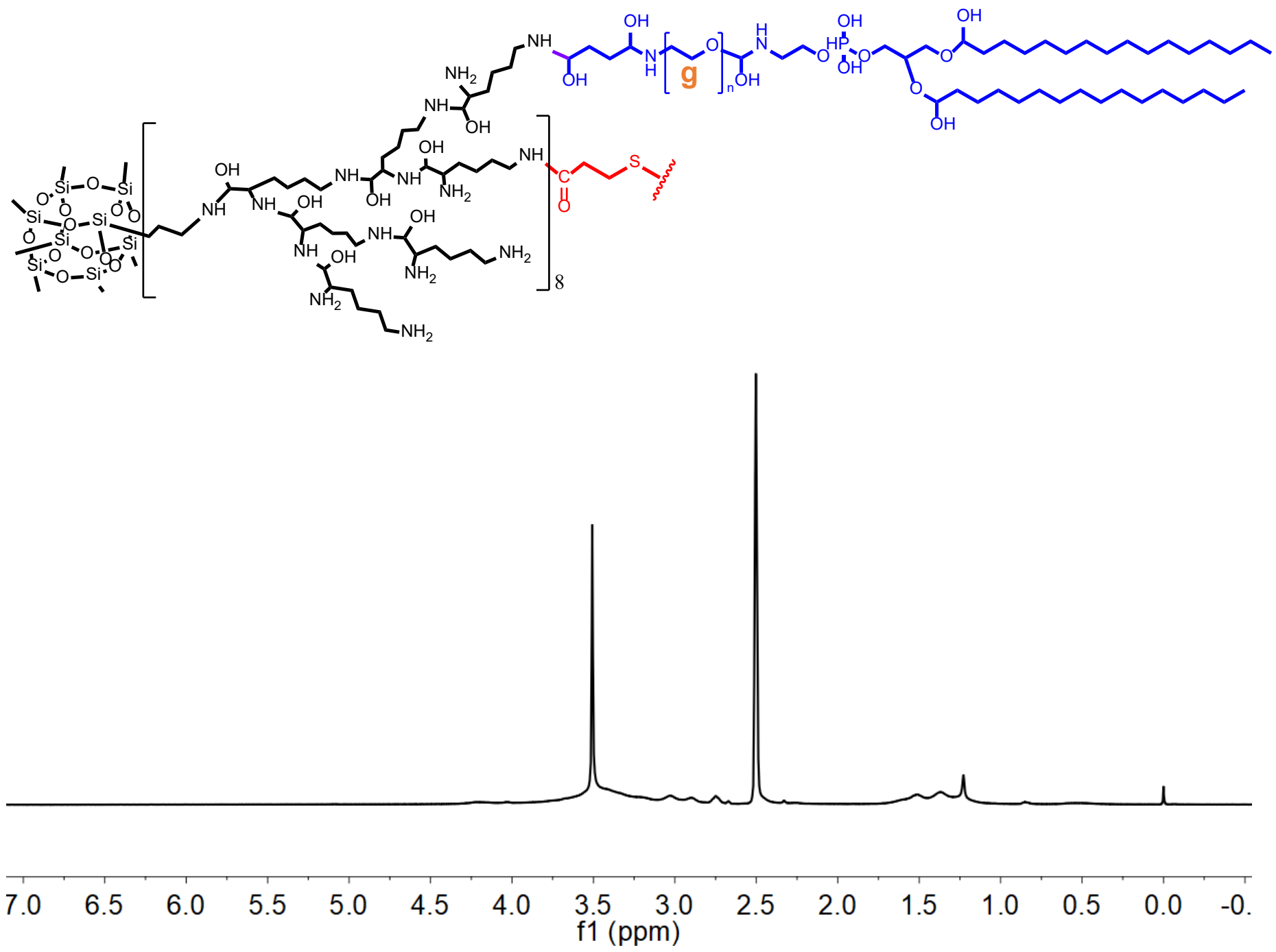

G3KDSP-DSPE

Figure S6. ^{1}H NMR spectrum of the resulting G3K-DSPE nanogels. The characteristic peaks from DSPE-PEG (δ ~3.6 ppm for PEG) confirm successful functionalization, with a calculated grafting ratio of 6.88%.

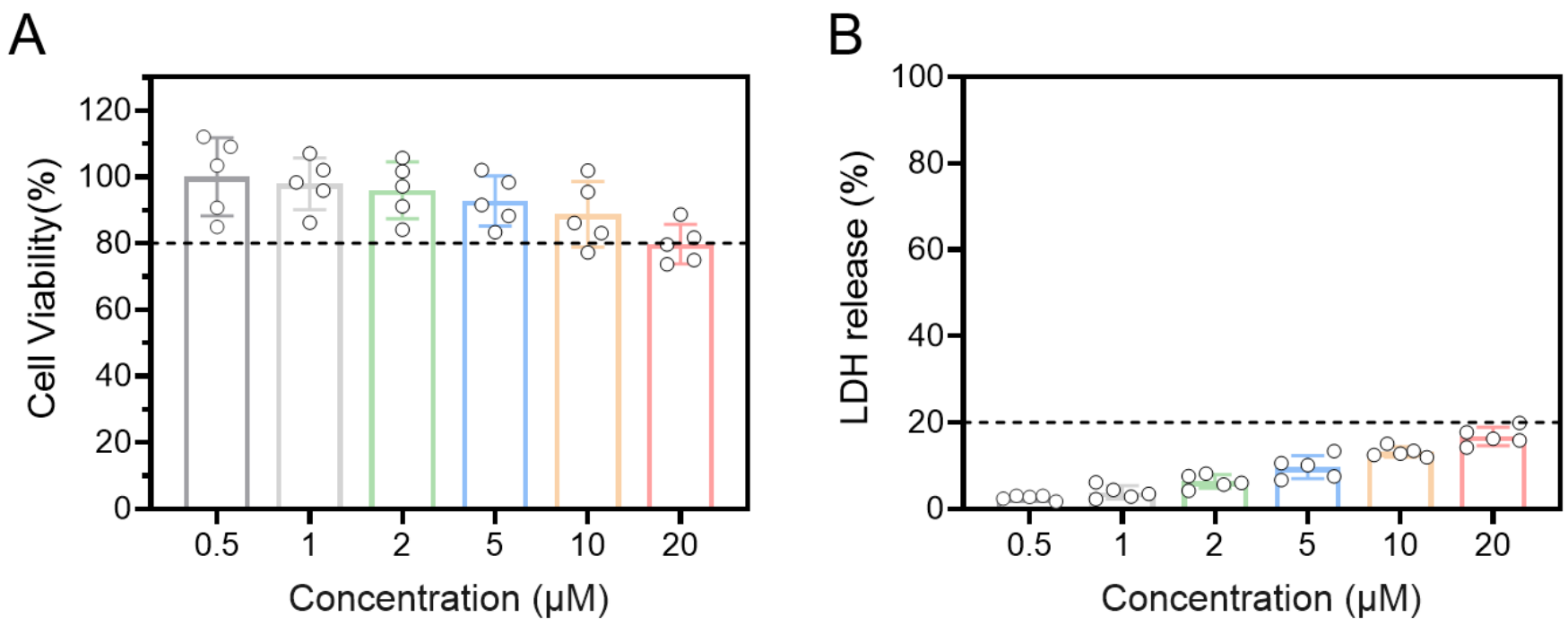

Figure S7. (A) Cell viability of MSCs after 24-hour incubation with varying concentrations of G3K@T nanogels, as assessed by CCK-8 assay. Viability remained above 80% across all tested concentrations. (B) Lactate dehydrogenase (LDH) release assay of MSCs after 24-hour incubation with varying concentrations of G3K@T nanogels. No significant increase in LDH release was observed across all tested concentrations compared to untreated MSCs, confirming that membrane-anchoring does not compromise membrane integrity. Data are mean ± SD (n = 5).

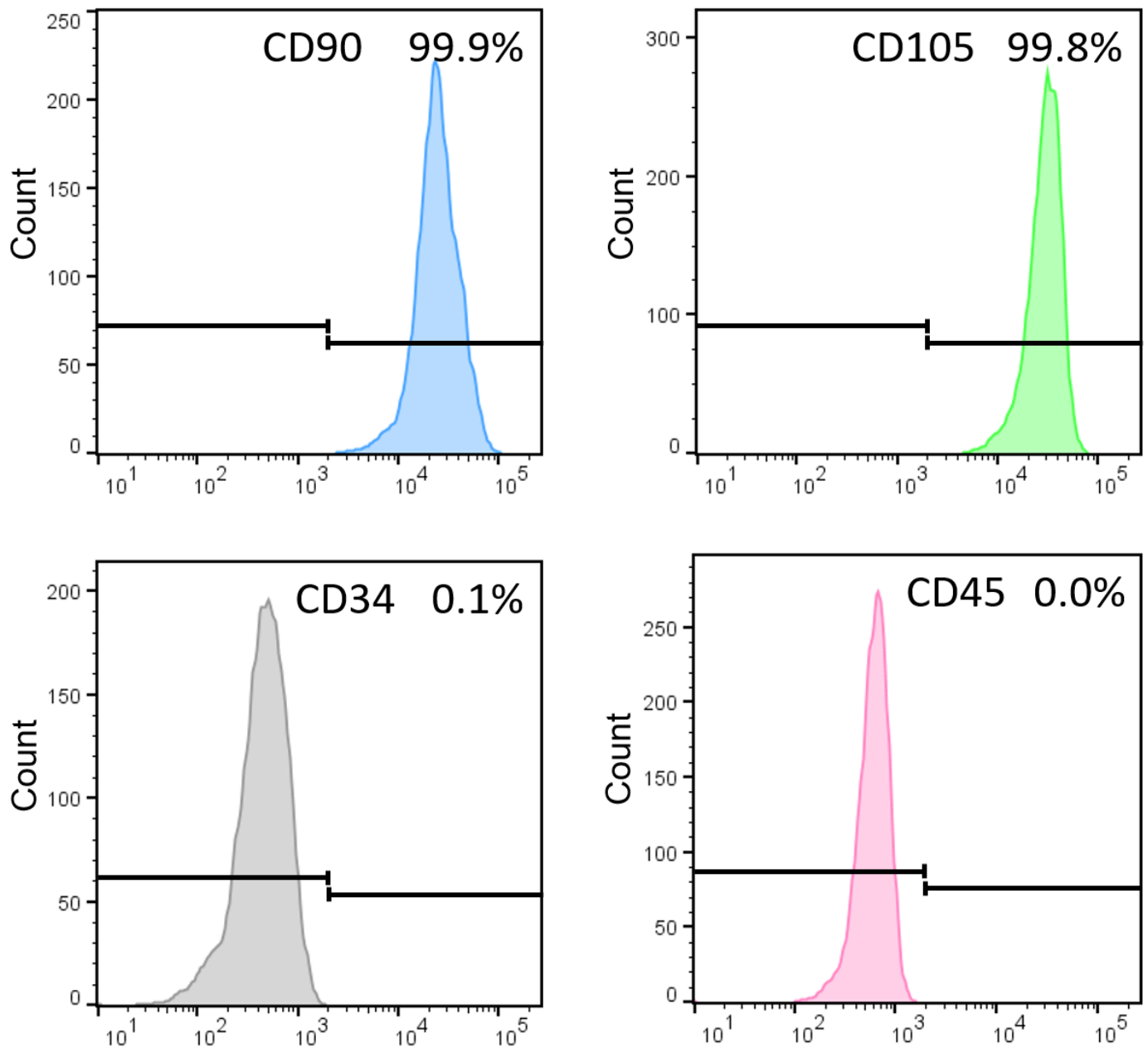

Figure S8. Flow cytometric analysis of MSC surface marker expression after G3K@T nanogel conjugation.

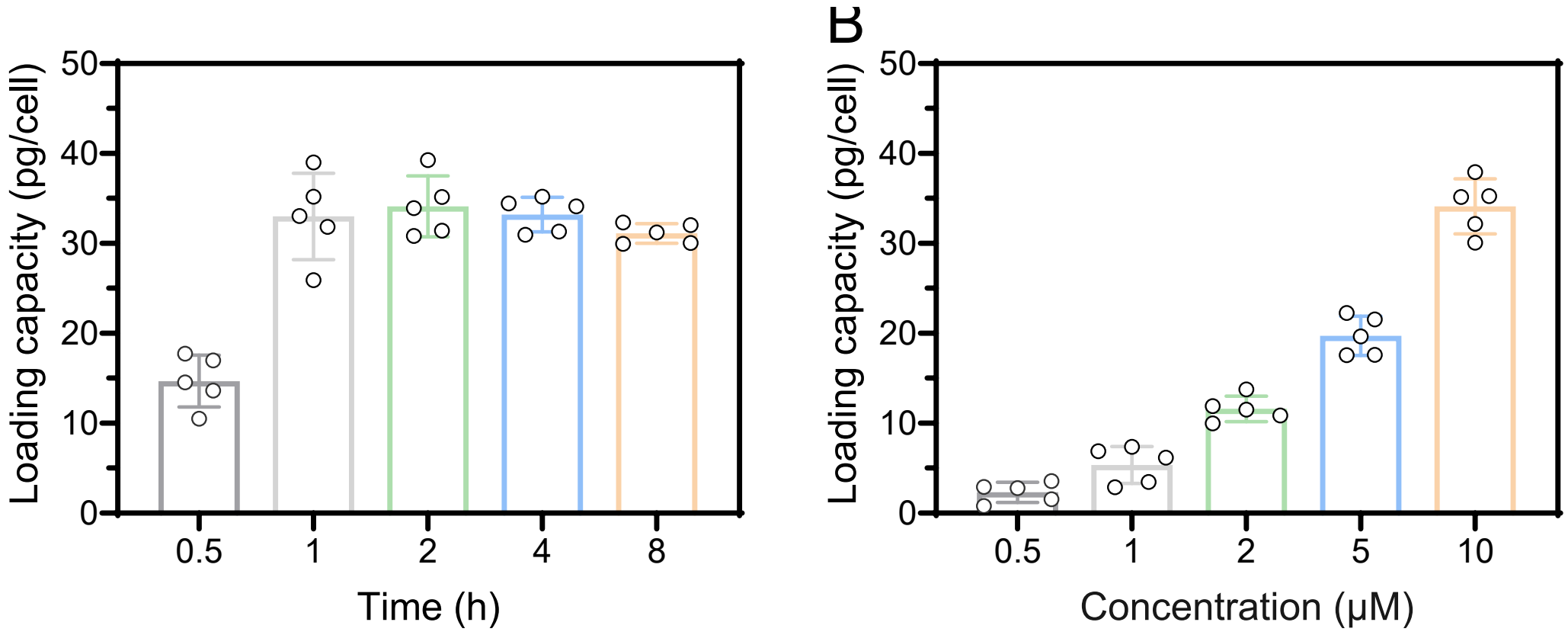

Figure S9. The tacrolimus loading capacity of MSCs incubated with G3K@T for different times (A) and at different concentrations (B). Data are presented as mean ± SD (n = 5).

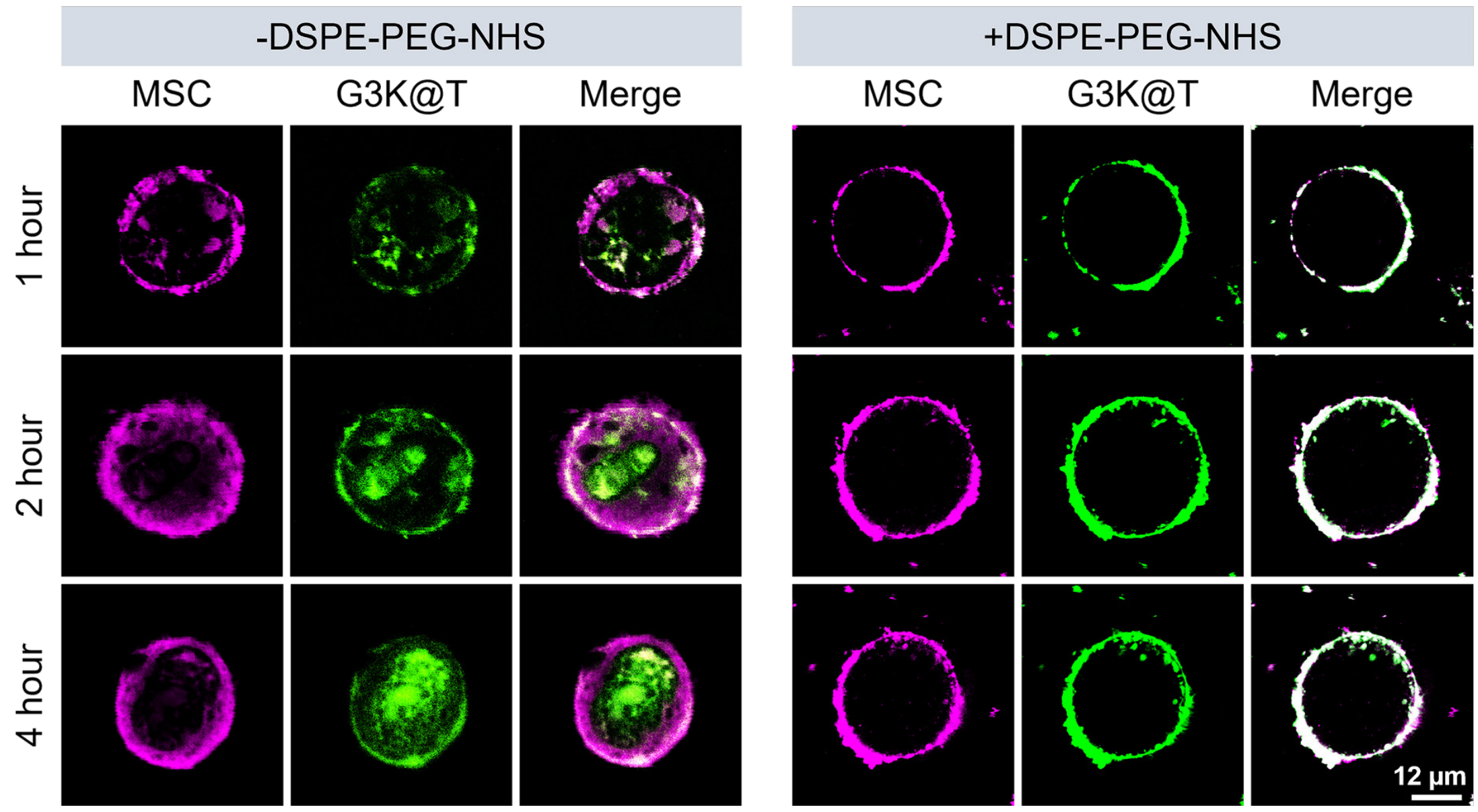

Figure S10. Representative confocal microscopy images of MSCs incubated with either FITC-labeled G3K@T (non-PEGylated, green) or G3K@T (DSPE-PEG-modified, green) for 1, 2 and 4 hours. MSCs were stained with CellMask orange (violet). Non-PEGylated nanogels show prominent intracellular accumulation over time, whereas PEGylated nanogels remain predominantly localized on the cell membrane.

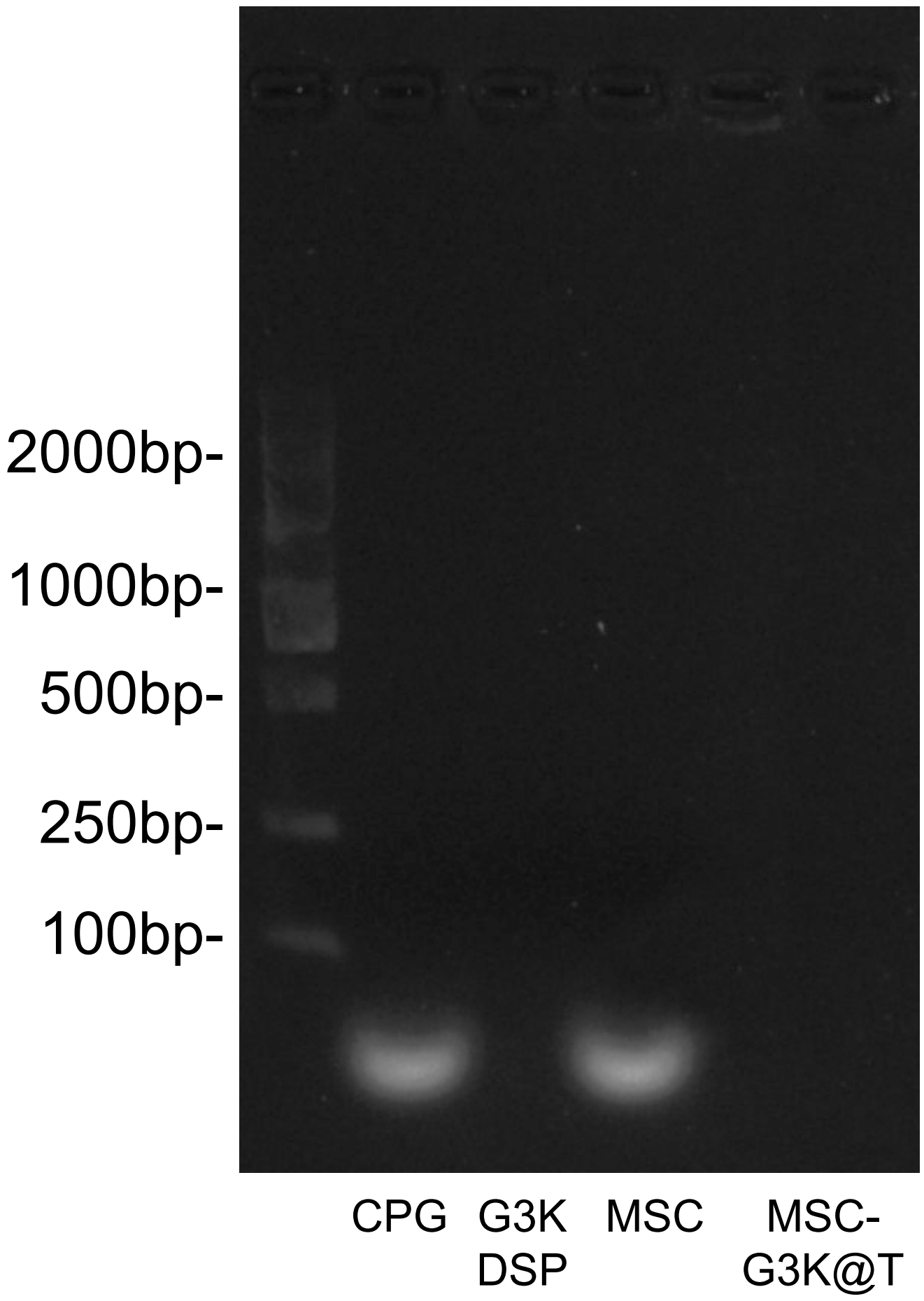

Figure S11. Agarose gel electrophoresis image showing DNA migration patterns. Lane 1: Free CpG-ODN2006 alone (control). Lane 2: CpG-ODN2006 incubated with G3KDSP nanogels. Lane 3: CpG-ODN2006 incubated with MSC. Lane 4: CpG-ODN2006 incubated with MSC-G3K@T. The complete absence of free DNA bands in lanes 2 and 4 indicates 100% binding efficiency of both the G3KDSP nanogels and the engineered cell platform.

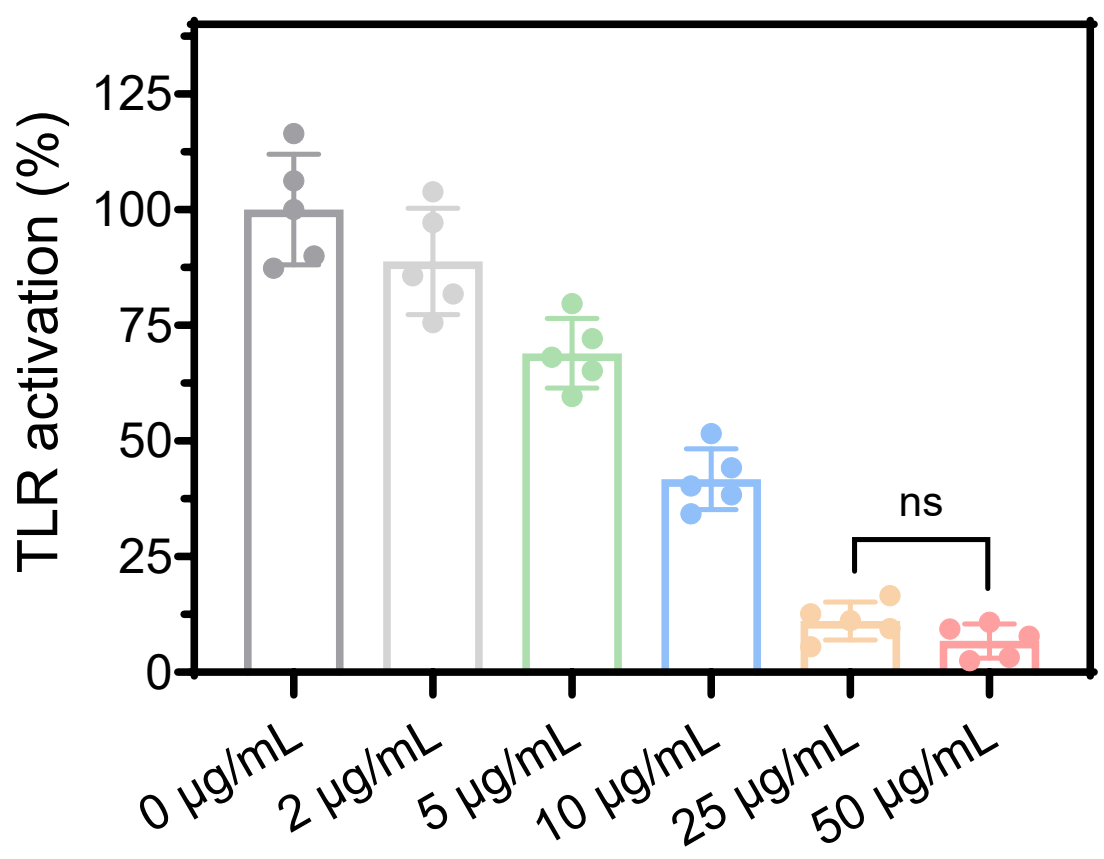

Figure S12. Ramos Blue™ reporter cells were stimulated with CpG-ODN2006 (TLR9 agonist) in the presence of MSC-G3K@T engineered with varying doses of conjugated G3K@T nanogels. Data are presented as mean ± SD (n = 5). Statistical significance was determined by one-way ANOVA with Tukey's post hoc test; ns, not significant.

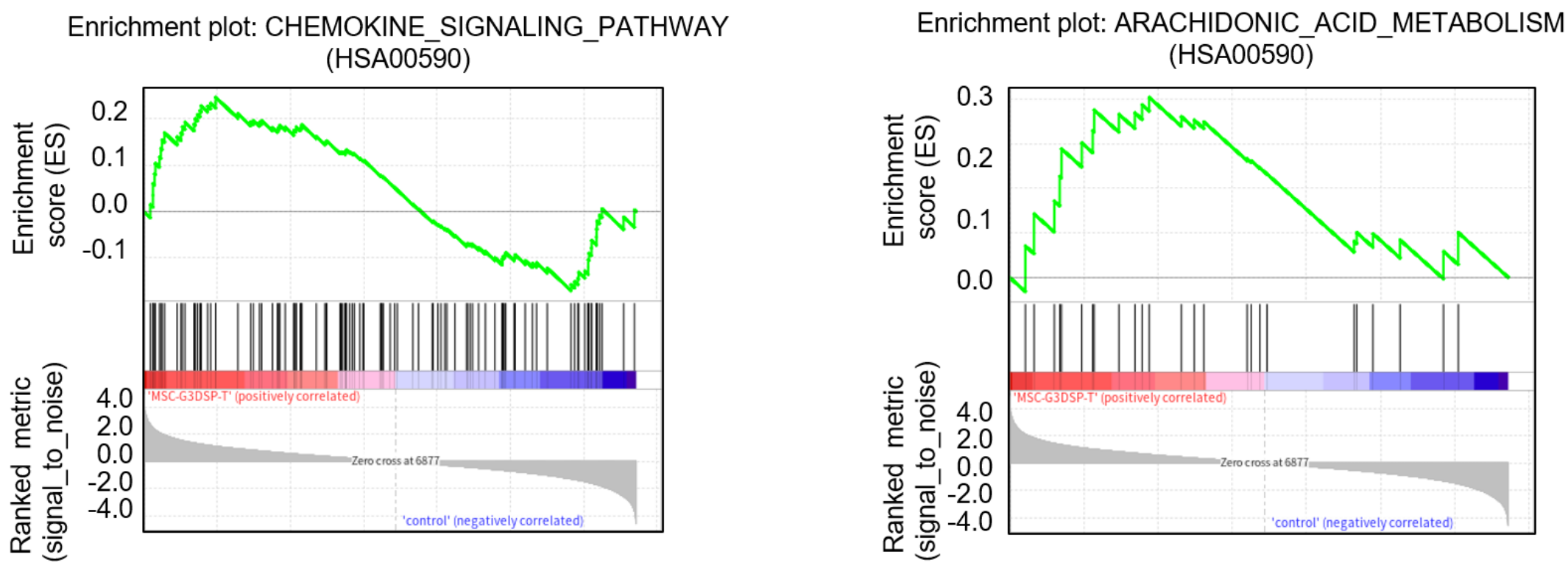

Figure S13. GSEA plots demonstrating significant enrichment of key pathways in MSC-G3K@T: Chemokine signaling and arachidonic acid metabolism.

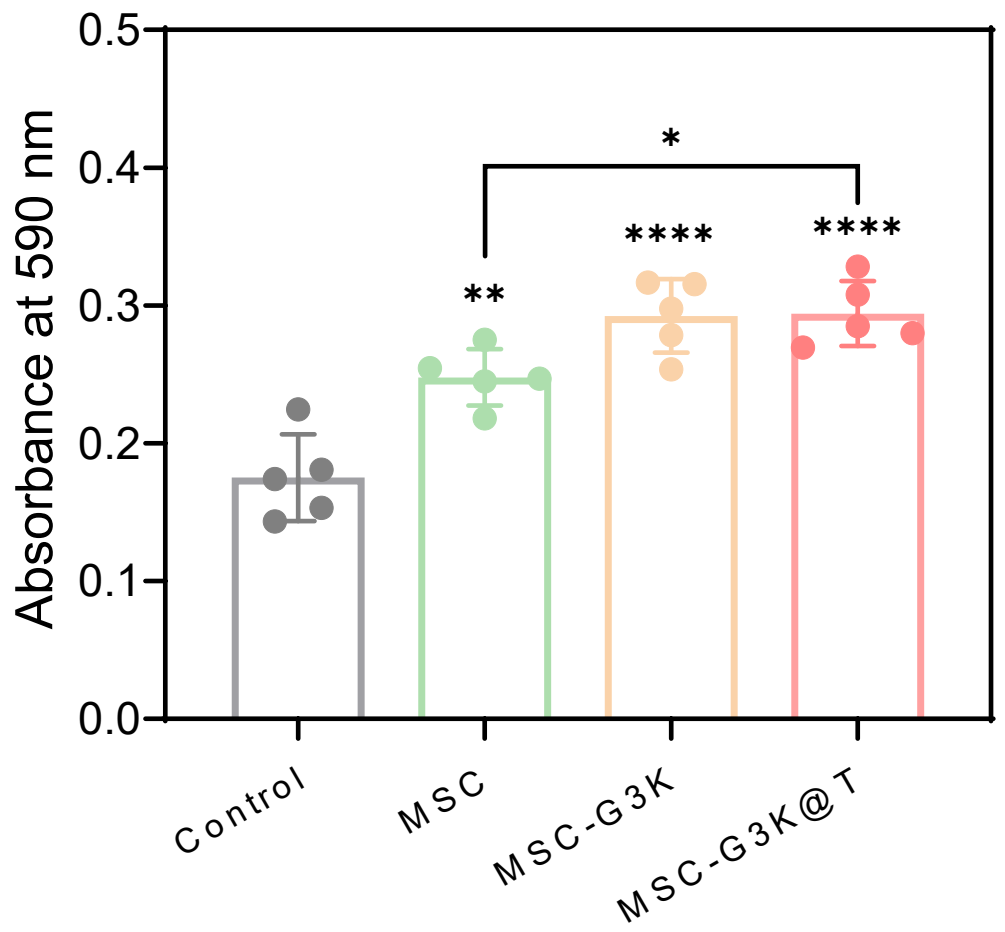

Figure S14. The quantification of migration assay toward CXCL12. Data are presented as mean ± SD (n = 5). Statistical significance was determined by one-way ANOVA with Tukey's post hoc test; *$p < 0.05$, **$p < 0.01$, ****$p < 0.0001$.

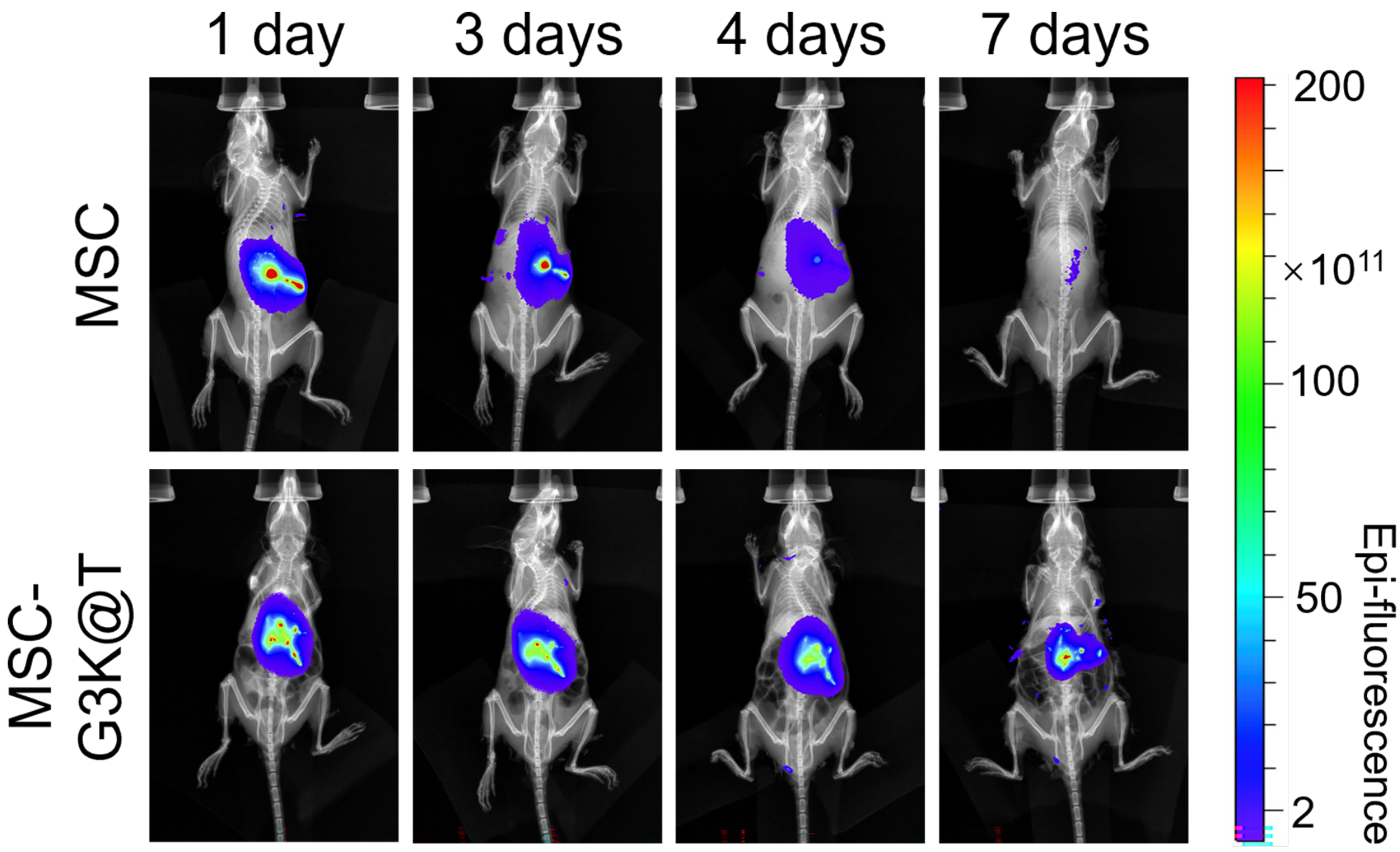

Figure S15. In vivo persistence of MSC-G3K@T in immunocompetent mice. DiD-labeled MSC-G3K@T or naïve MSCs were subcutaneously implanted in BALB/c mice, and fluorescence signal was monitored over 7 days.

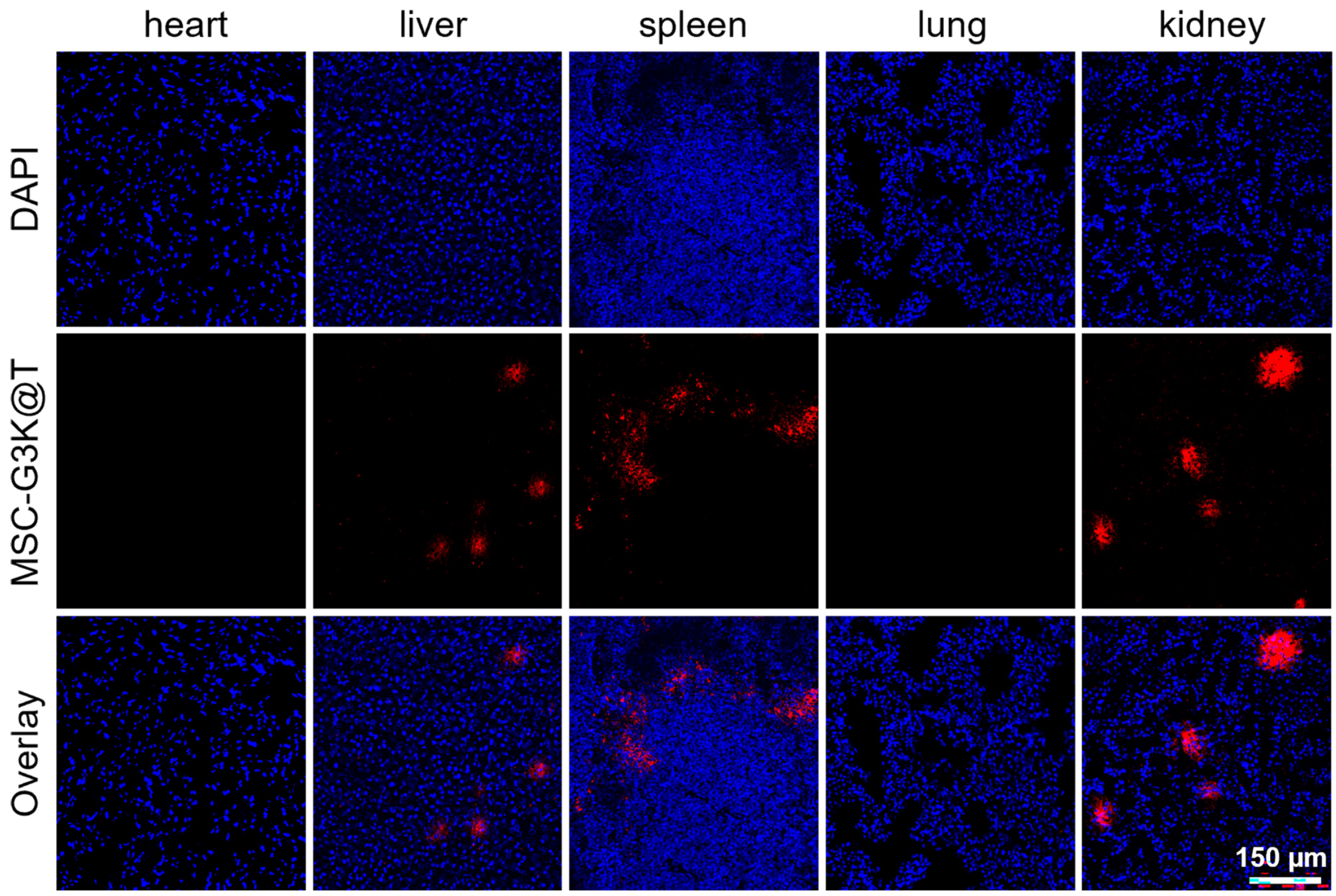

Figure S16. Representative fluorescence images of heart, liver, spleen, lung, and kidney sections from treated mice.

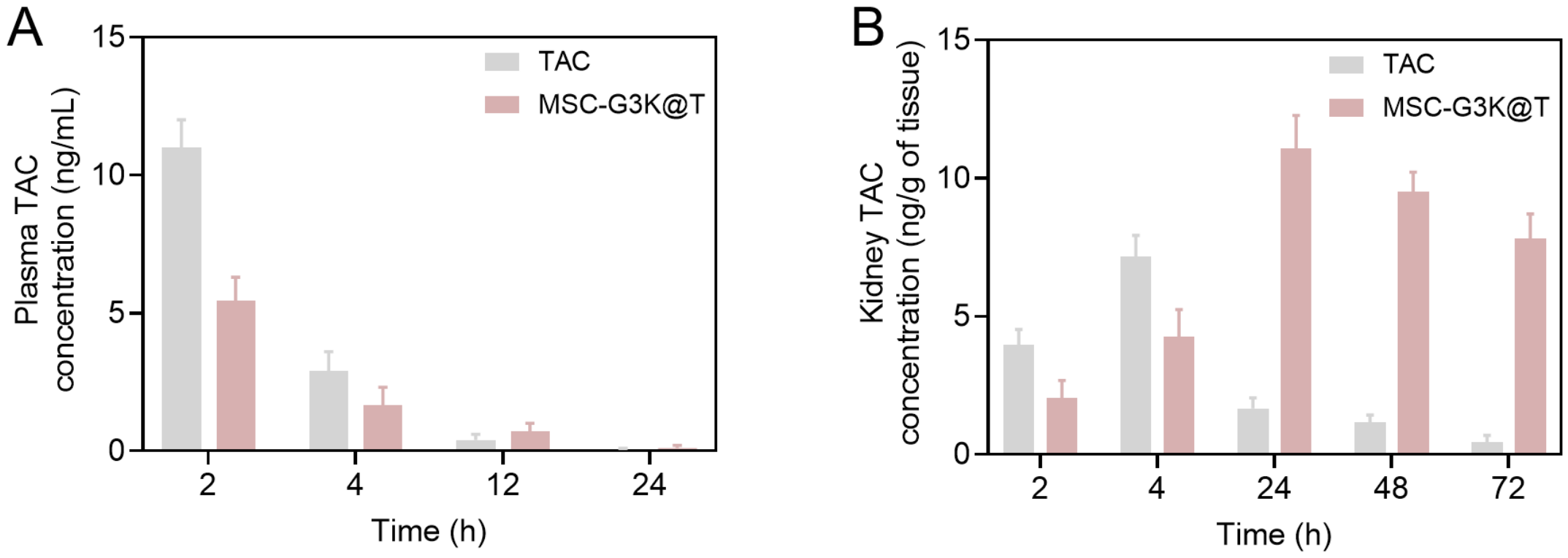

Figure S17. Pharmacokinetic analysis of tacrolimus after MSC-G3K@T administration. (A) Plasma tacrolimus concentration following intravenous injection of MSC-G3K@T or an equivalent dose of free tacrolimus in MRL/lpr mice within 24 hours. (B) Renal tacrolimus concentrations at 2, 4, 24, 48, and 72 h post-injection. MSC-G3K@T significantly prolonged the renal retention and increased renal drug exposure compared to free tacrolimus. Data are presented as mean ± SD (n = 3).

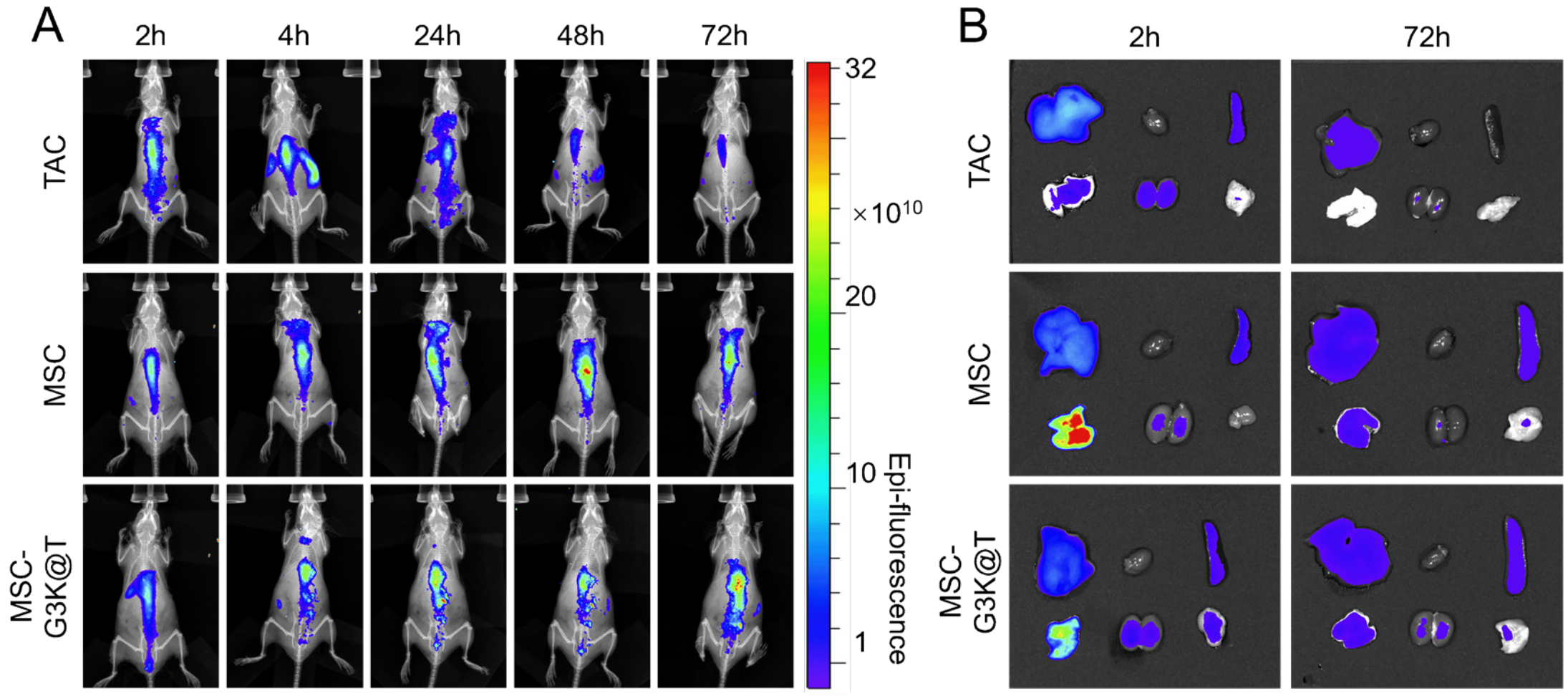

Figure S18. Renal targeting specificity evaluated in healthy MRL/MpJ mice. (A) In vivo fluorescence imaging of DiD-labeled MSC-G3K@T in MRL/MpJ mice mice at 2, 4, 24, 48, 72 h post-injection and (B) Ex vivo fluorescence imaging of DiD-labeled MSC-G3K@T in MRL/MpJ mice at 2 and 72 h post-injection, compared with MSC-G3K@T and free Cy5-tacrolimus in MRL/lpr mice.

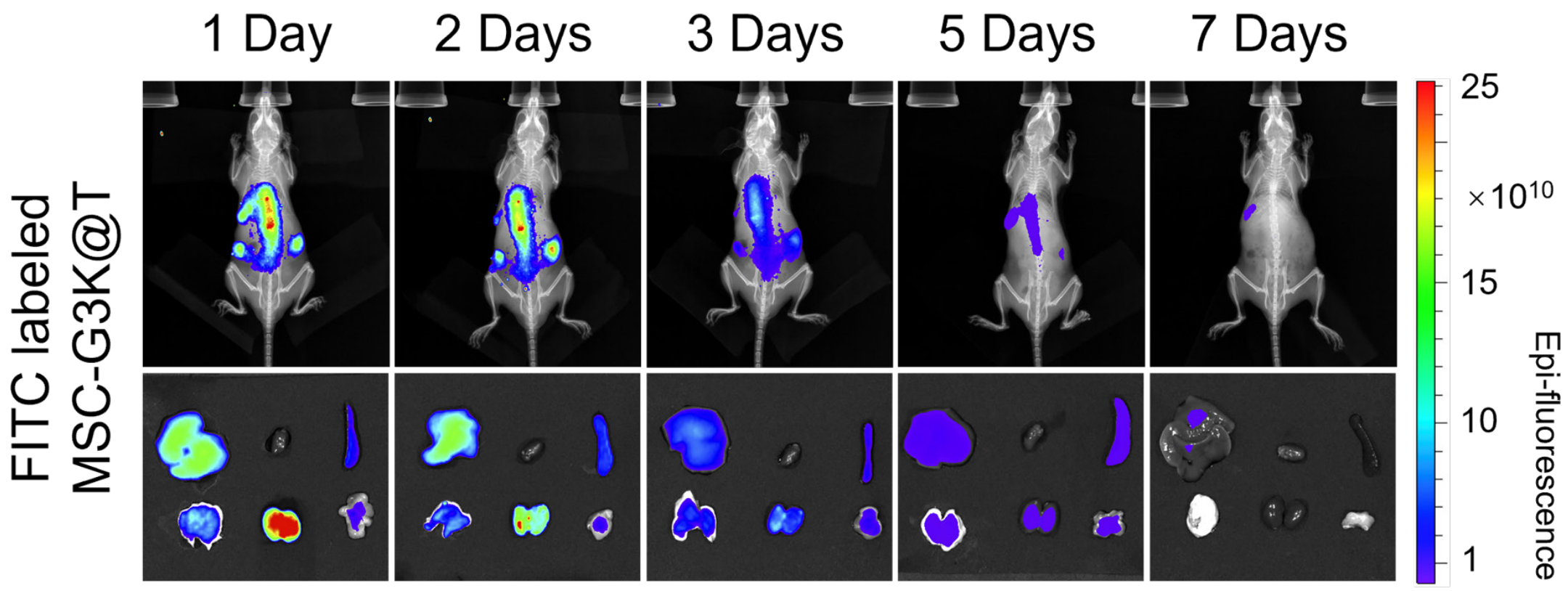

Figure S19. In vivo and Ex vivo fluorescence images of renal FITC signal at 1, 2, 3, 5, and 7 days after intravenous injection of MSC-G3K@T carrying FITC-labeled G3K nanogels in MRL/lpr mice. The renal fluorescence intensity gradually declined and returned to near-background by day 7, indicating efficient clearance of the nanogel component without chronic sequestration.

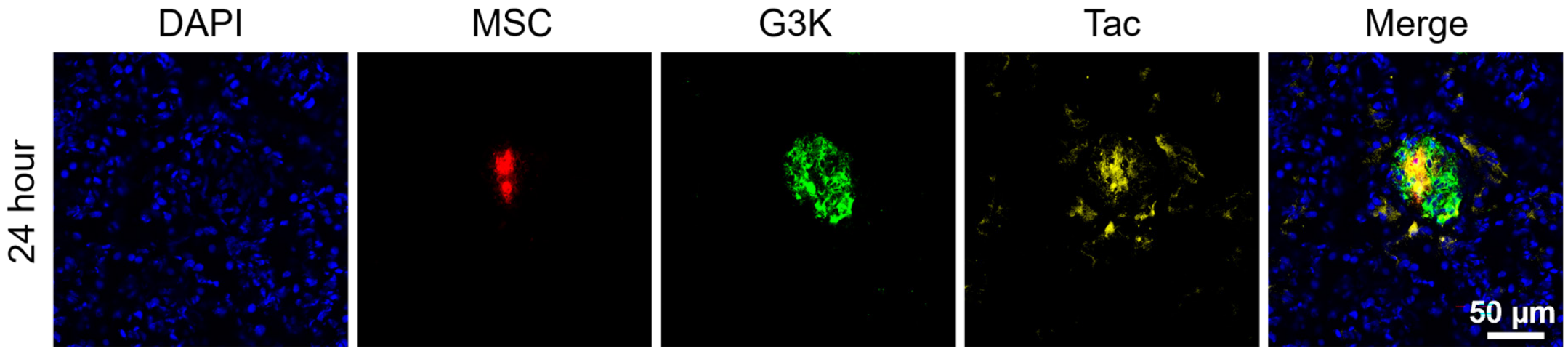

Figure S20. Validation of ROS-dependent drug release in healthy MRL/MpJ mice. Triple-labeled MSC-G3K@T (MSC: CellMask orange, red; G3KDSP nanogel: FITC, green; tacrolimus: Cy5, yellow) was intravenously injected into MRL/MpJ mice. Confocal images of kidney sections at 24 h post-injection show that tacrolimus and nanogel signals remain tightly co-localized with the MSC membrane.

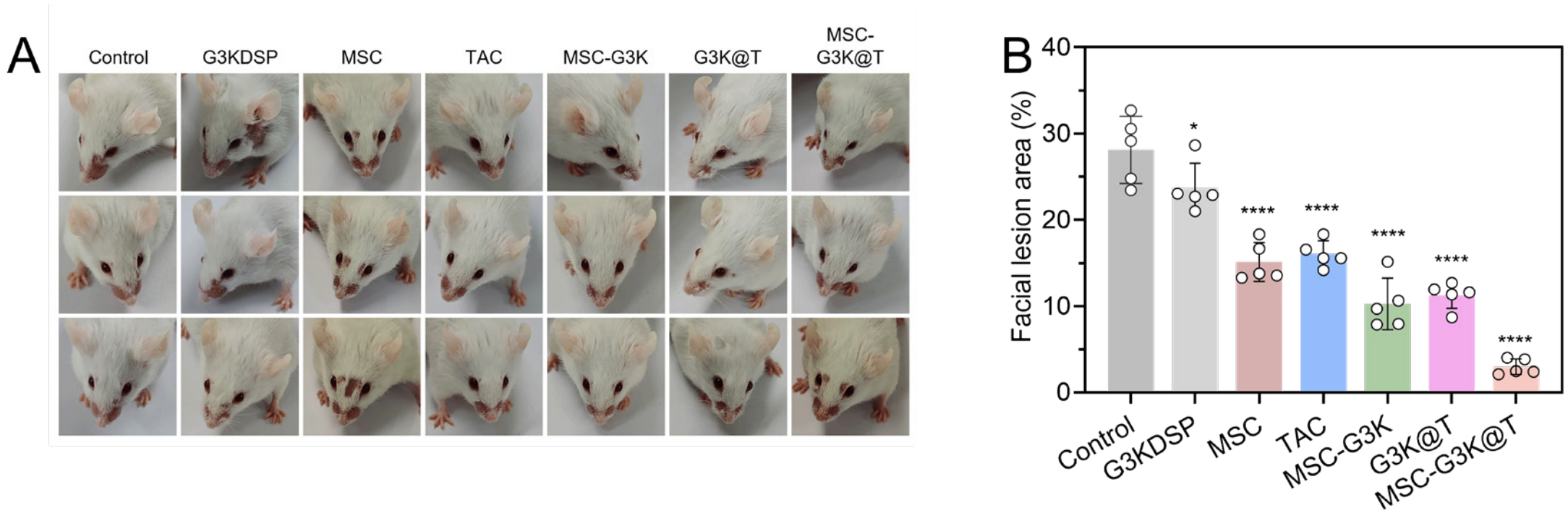

Figure S21. Assessment of skin lesion severity in MRL/lpr mice. (A) Representative photographs of facial skin lesions before treatment with saline, G3KDSP nanogels, MSCs, tacrolimus, MSC-G3K, G3K@T, or MSC-G3K@T. (B) Quantification of visible skin lesion area from standardized photographs using ImageJ, performed by an observer blinded to treatment groups. MSC-G3K@T treatment significantly reduced skin lesion area compared to all other groups. Data are mean ± SD (n = 5). Statistical significance was determined by one-way ANOVA with Tukey's post hoc test; *$p < 0.05$, ****$p < 0.0001$.

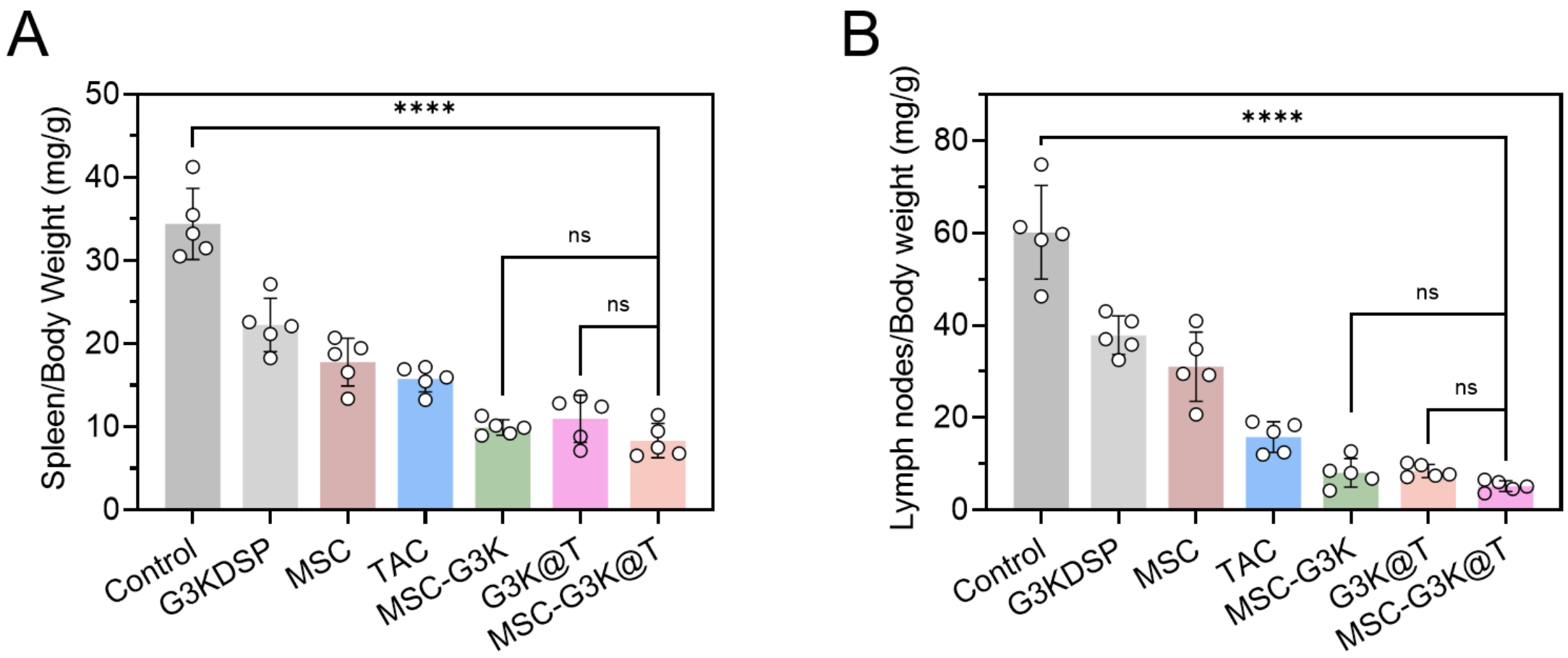

Figure S22. Quantitative analysis of (A) spleen-to-body-weight ratio and (B) major lymph nodes-to-body-weight ratio per group. Data are presented as mean ± SD (n = 5). ****p < 0.0001, ns, not significant versus saline-treated control group (one-way ANOVA with Tukey's post-hoc test).

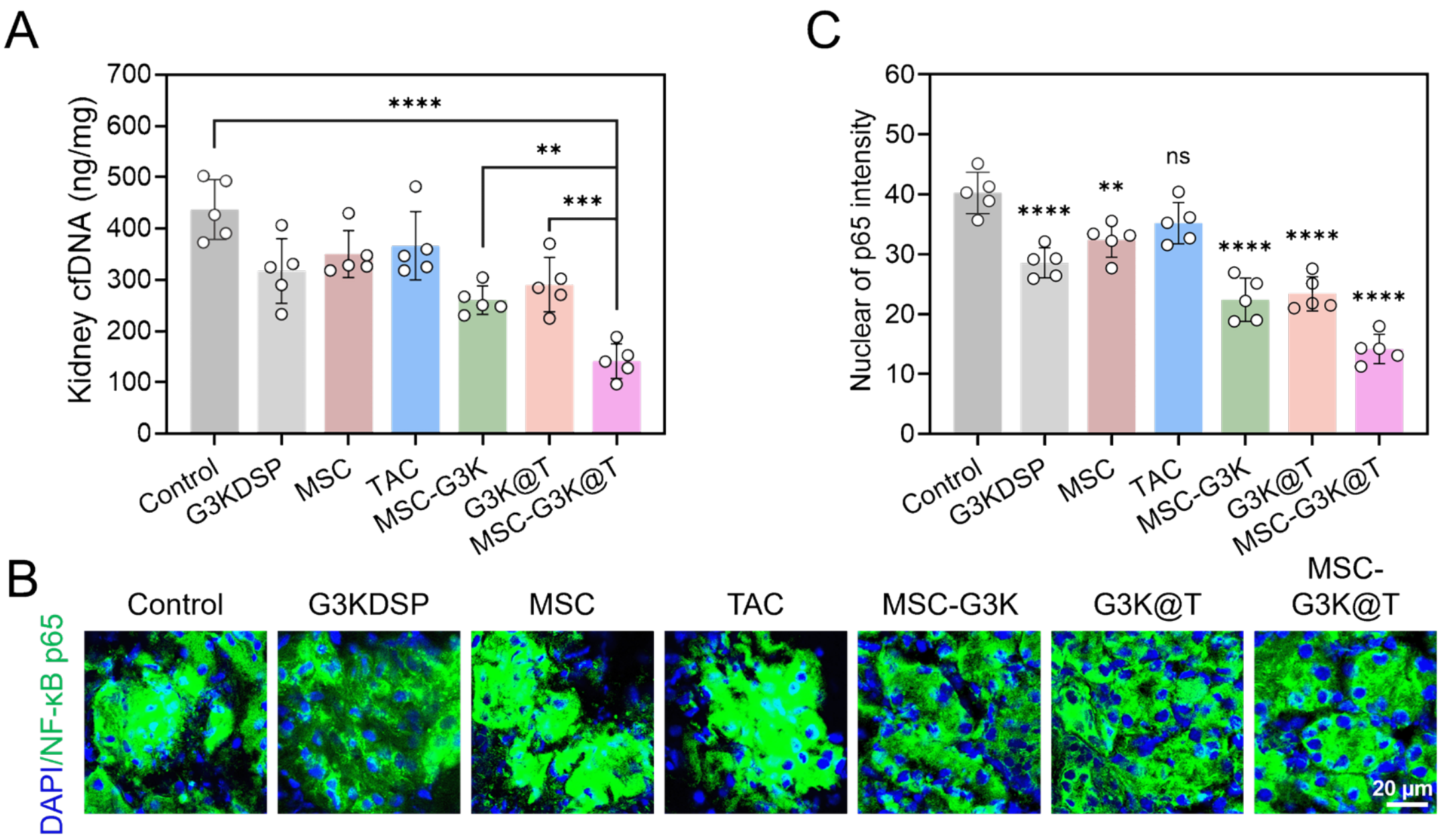

Figure S23. In vivo cfDNA clearance and NF-κB p65 suppression in kidney tissues. (A) Quantification of cfDNA levels in kidney tissue homogenates from MRL/lpr mice after treatment with saline, G3KDSP nanogels, MSCs, tacrolimus, MSC-G3K, G3K@T, or MSC-G3K@T. (B) Representative immunofluorescence images of NF-κB p65 (green) expression in kidney sections. DAPI (blue) was used for nuclear counterstaining. Data are presented as mean ± SD (n = 5). Statistical significance was determined by one-way ANOVA with Tukey's post hoc test; $^{**}p < 0.01$, $^{***}p < 0.001$, $^{****}p < 0.0001$, ns, not significant.

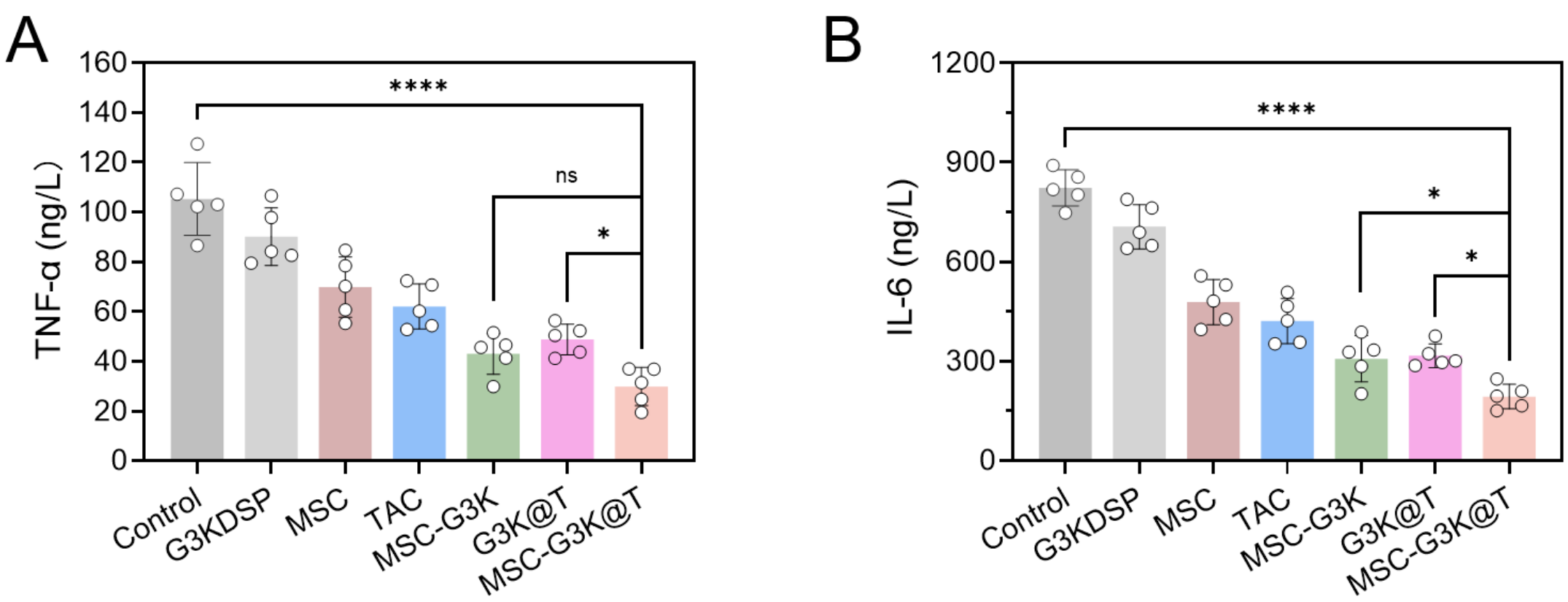

Figure S24. Quantification of (A) TNF-α and (B) IL-6 serum by ELISA after four-week treatment. Data are presented as mean ± SD (n = 5). *p < 0.05, ****p < 0.0001, ns, not significant vs. control group (one-way ANOVA with Tukey's post-hoc test).

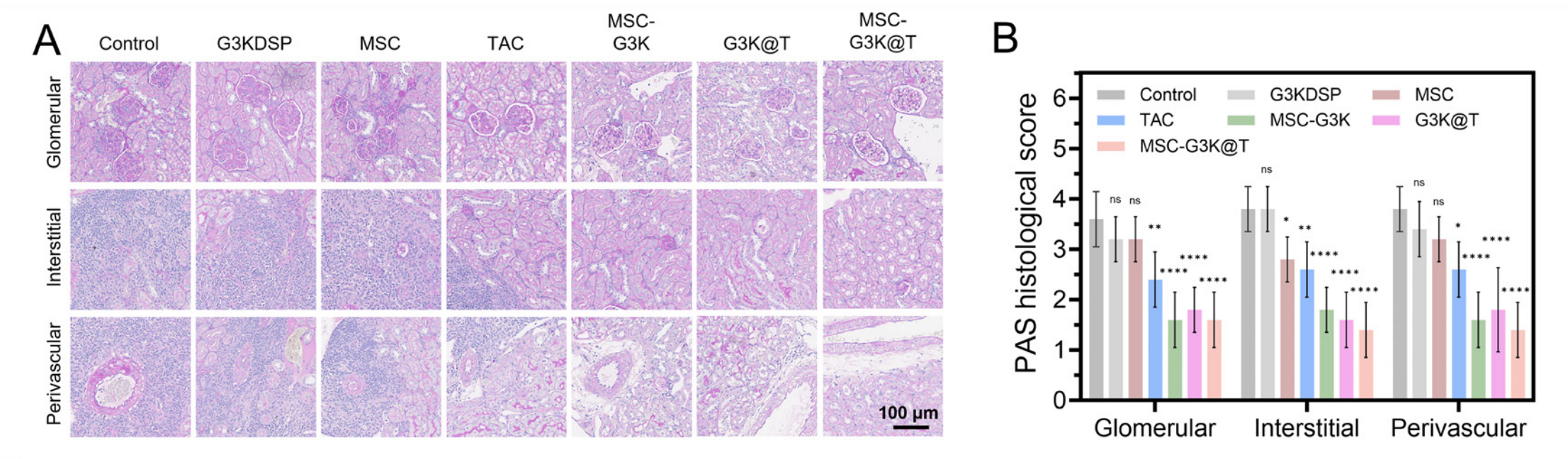

Figure S25. (A) Representative images of kidney sections stained with PAS and (B) Quantitative histopathological scores of glomerular, interstitial, and perivascular inflammations from PAS staining. Data are mean ± SD (n = 5). *p < 0.05, **p < 0.01, ****p < 0.0001, ns, not significant versus saline-treated control group (one-way ANOVA with Tukey's post-hoc test).

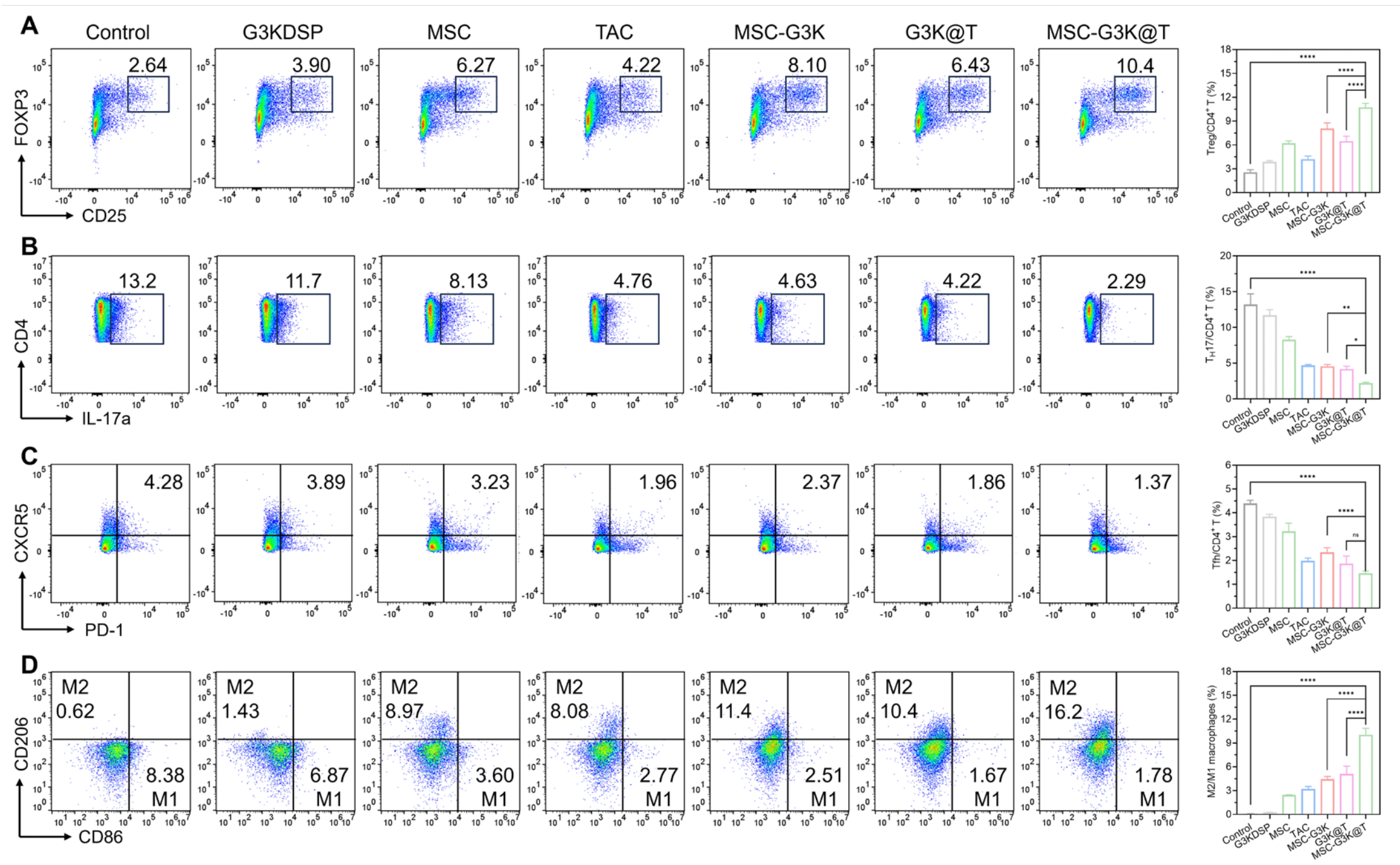

Figure S26. MSC-G3K@T Systemically Reshapes the Immune Profile in Peripheral Blood of MRL/lpr Mice. (A) Flow cytometric analysis and quantification of peripheral blood regulatory T cells (Tregs, $CD4^{+}FOXP3^{+}$). (B, C) Analysis of peripheral blood pro-inflammatory T cell subsets: (B) frequency of $T_{H}17$ cells ($CD4^{+}IL\text{-}17A^{+}$), (C) frequency of T follicular helper cells (Tfh, $CD4^{+}CXCR5^{+}PD\text{-}1^{+}$). (D) Quantification of peripheral blood pro-inflammatory neutrophils ($Ly\text{-}6G^{+}CD11b^{+}$). (E) Analysis of peripheral blood macrophage polarization, presented as the M2/M1 ratio based on $CD206^{+}$ (M2) and $CD86^{+}$ (M1) expression among $CD68^{+}$ monocytes/macrophages. All data are derived from PBMCs and correspond to the splenic immune analyses shown in Figure 9. Values are presented as mean ± SD (n = 5). Statistical significance was determined by one-way ANOVA with Tukey's post-hoc test; *$p < 0.05$, **$p < 0.01$, ****$p < 0.0001$, not significant.

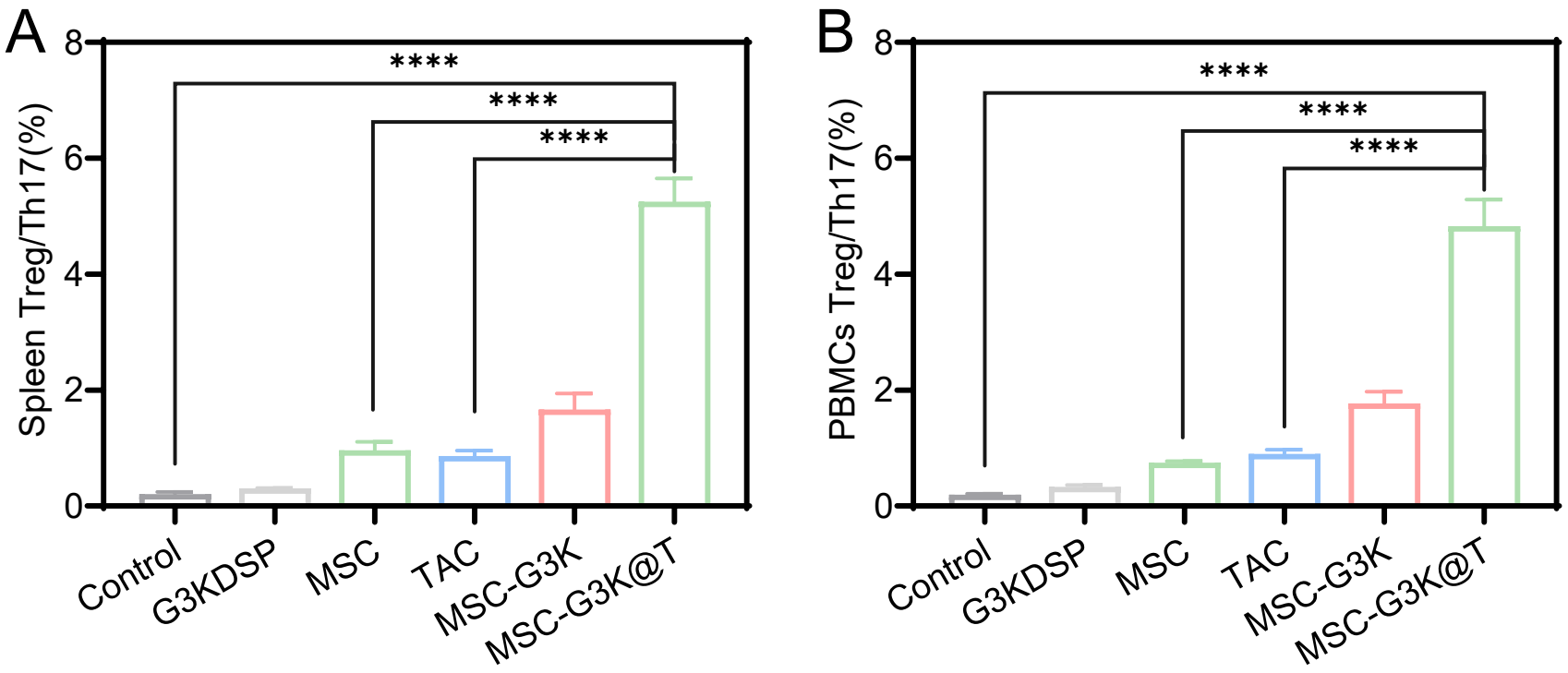

Figure S27. Quantification of the Treg/T_H17 cell ratio in (A) splenocytes and (B) PBMCs from each treatment group. A higher ratio indicates a shift toward immune tolerance. Data are mean ± SD (n = 5). ****$p < 0.0001$ versus control (one way ANOVA with Tukey's post hoc test).

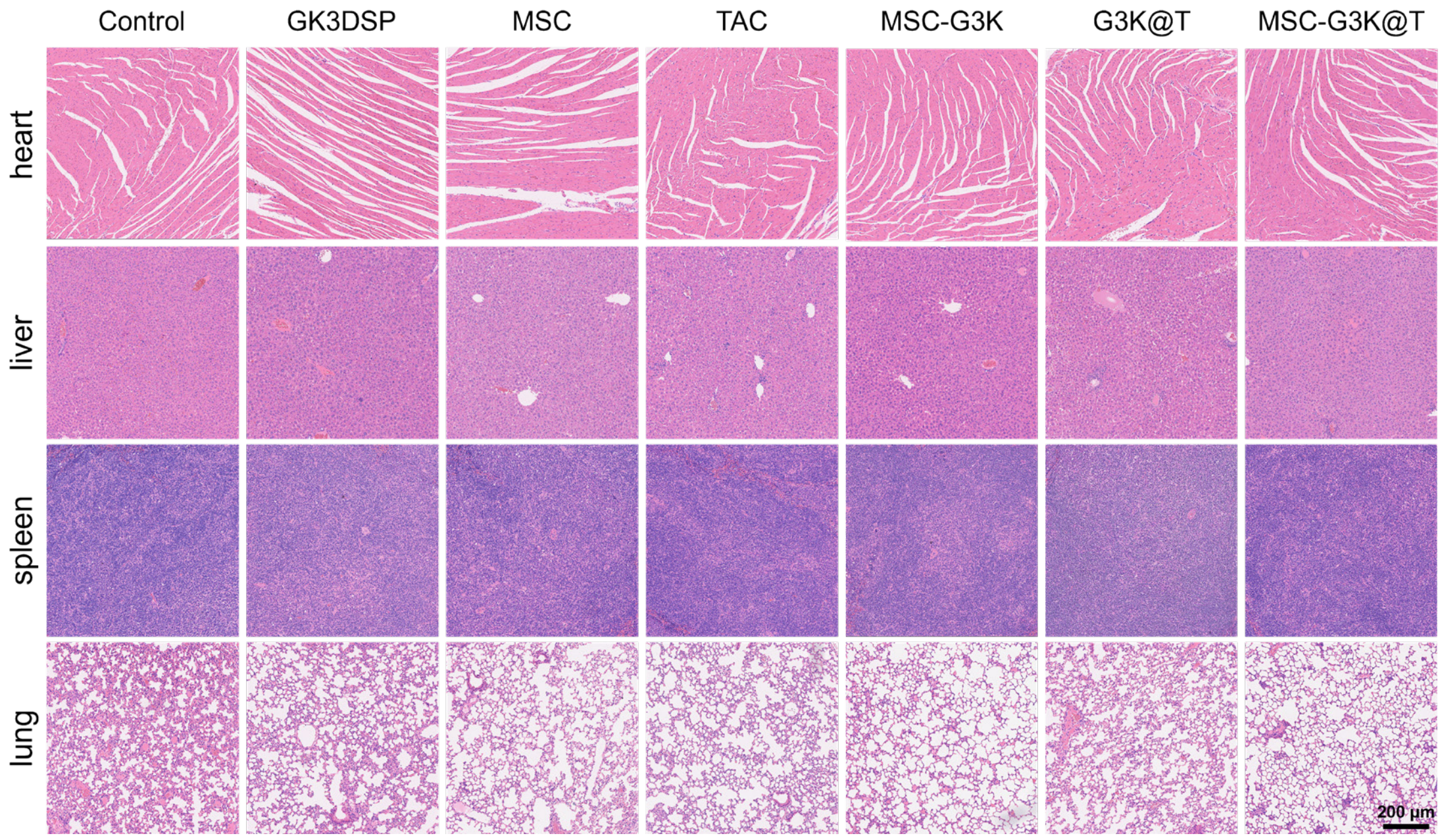

Figure S28. Representative H&E-stained sections of heart, liver, spleen, and lung from each group. No significant pathological changes attributable to treatment were observed.

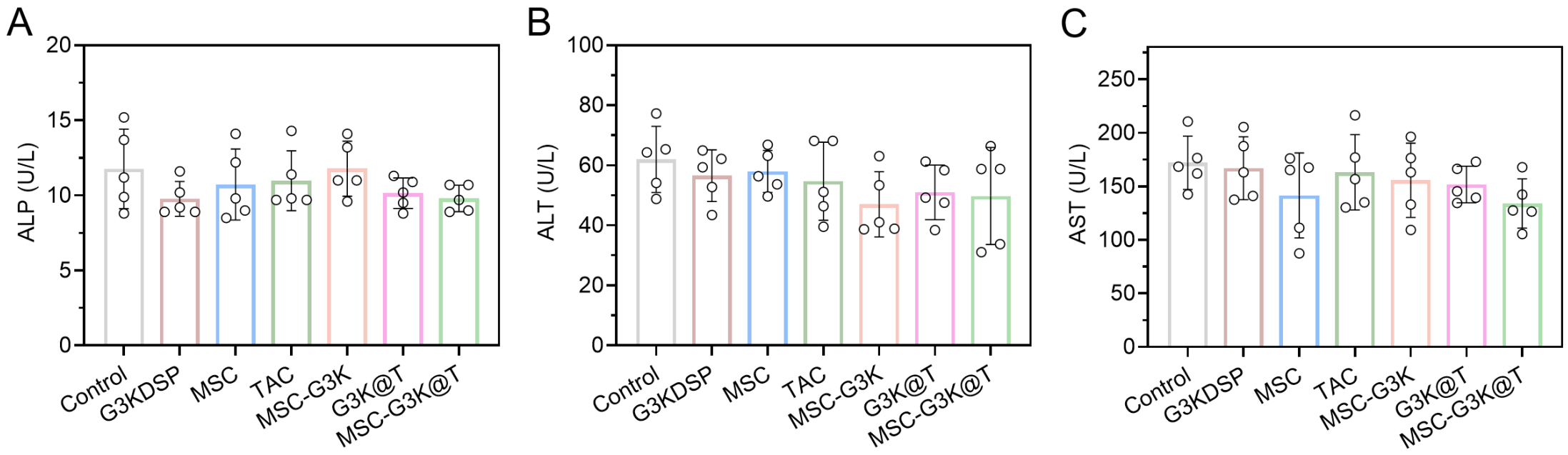

Figure S29. Serum levels of hepatic function markers. (A) ALP, (B) ALT, and (C) AST. All measured values fell within the normal reference intervals. Data are mean ± SD (n = 5).

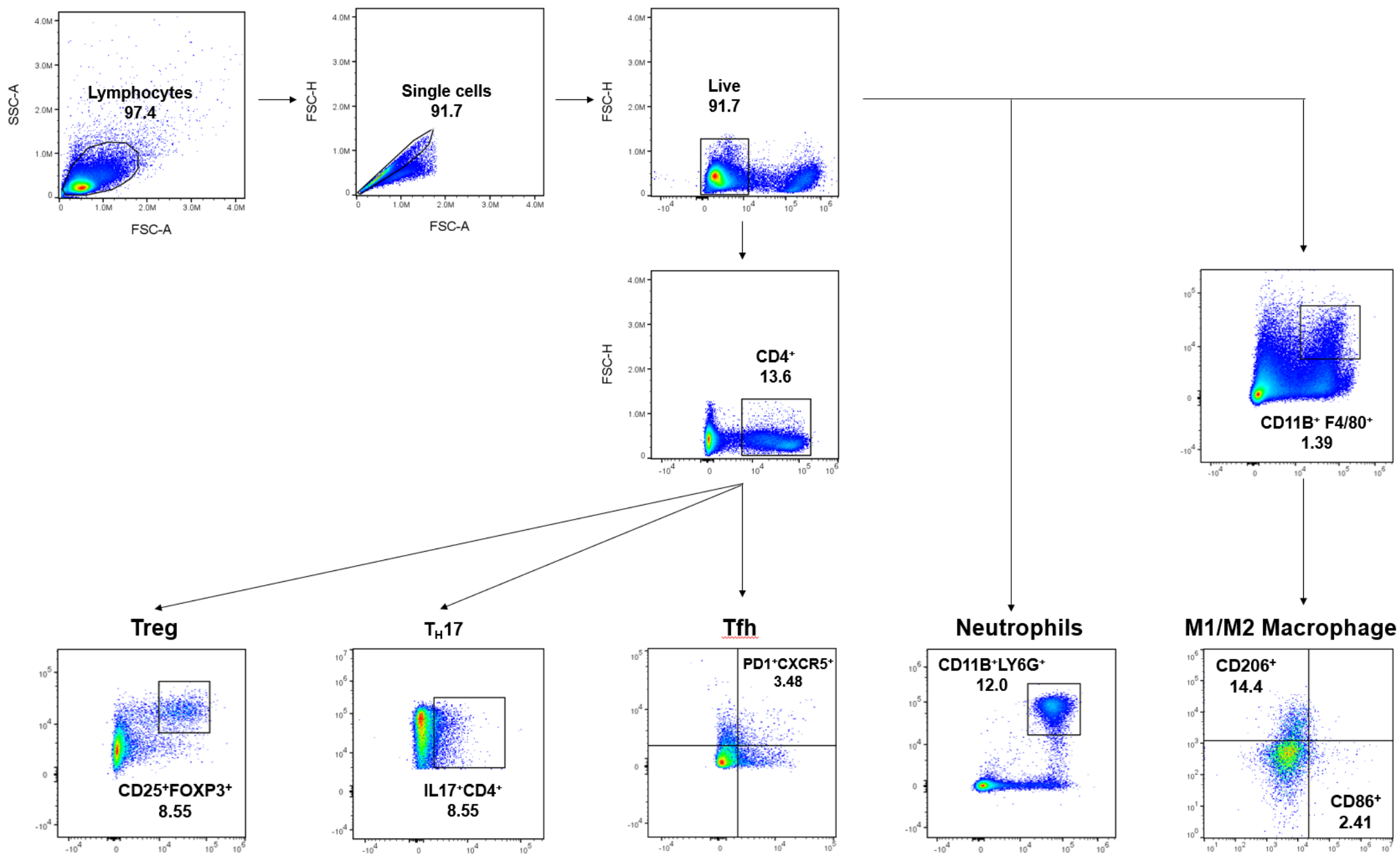

Figure S30. The gating strategy for flow cytometry.

Table S1. Bliss independence synergy analysis of MSC and G3K@T nanogel combination therapy. Synergy scores were calculated as the ratio of the observed effect of MSC-G3K@T to the predicted effect for proteinuria, renal cfDNA level, serum anti-dsDNA antibody, CRE, BUN and TNF-α. A score >1 indicates synergy.

	E_{MSC}	$E_{G3K@T}$	E_{Bliss}	E_{EXP}	Synergy Score (E_{EXP} / E_{Bliss})
**Proteinuria**	0.284	0.317	0.511	0.553	1.082
**cfDNA**	0.157	0.529	0.603	0.734	1.217
**Anti-dsDNA**	0.309	0.462	0.628	0.638	1.016
**CRE**	0.341	0.496	0.667	0.673	1.009
**BUN**	0.314	0.522	0.672	0.731	1.086
**TNF-α**	0.336	0.536	0.692	0.716	1.098

$E_{Bliss} = E_{MSC} + E_{G3K@T} - E_{MSC} \times E_{G3K@T}$.
